# Comparative efficacy of incretin drugs on glycemic control, body weight, and blood pressure in adults with overweight or obesity and with/without type 2 diabetes: a systematic review and network meta-analysis

**DOI:** 10.3389/fendo.2025.1513641

**Published:** 2025-02-04

**Authors:** Song Liu, Jiaqiang Hu, Chen Zhao, Hang Liu, Chunyang He

**Affiliations:** ^1^ Department of Pharmacy, The Third People’s Hospital of Chengdu, The Affiliated Hospital of Southwest Jiao Tong University, Chengdu, Sichuan, China; ^2^ Department of Pharmacy, Guang’an People’s Hospital, Guang’an, Sichuan, China; ^3^ Department of Pharmacy, Nanchong Central Hospital, The Second Clinical Medical School of North Sichuan Medical College, Nanchong, Sichuan, China; ^4^ Department of Neurology, Affiliated Banan Hospital of Chongqing Medical University, Chongqing, China

**Keywords:** GLP-1 receptor agonists, network meta-analysis, multi-receptor drugs, obesity, type 2 diabetes

## Abstract

**Background:**

The rapid development of multi-receptor drugs targeting glucagon-like peptide-1 receptor (GLP-1R) is driving significant advancements in the treatment of individuals with type 2 diabetes and obesity. This systematic review and network meta-analysis aims to compare the efficacy and safety of multi-receptor drugs in adults with overweight or obesity, with or without type 2 diabetes.

**Methods:**

A systematic search was conducted in PubMed, Cochrane, Web of Science, Embase, CNKI, and WanFang databases up to May 12, 2024. Randomized controlled trials (RCTs) with an intervention duration of at least 12 weeks were included. The population of interest consisted of individuals with overweight or obesity, with or without type 2 diabetes. Eligible studies compared multi-receptor drugs with placebo or other multi-receptor drugs. The primary outcomes were weight reduction, glycated hemoglobin (HbA_1c_), fasting plasma glucose (FPG), blood pressure changes, and adverse events. Risk of bias was assessed using the version 2 of the Cochrane risk-of-bias tool (ROB2), and a random-effects network meta-analysis was performed using the frequentist approach. Confidence in effect estimates was evaluated using the Confidence In Network Meta-Analysis (CINeMA) framework.

**Results:**

A total of 24 trials, involving 9165 participants, were included. Retatrutide (mean difference (MD): -11.91 kg, 95% CI: -19.00 to -4.82, P-score: 0.80, p: 0.0003) and Tirzepatide (MD: -12.78 kg, 95% CI: -16.10 to -9.46, P-score: 0.89, p < 0.0001) exhibited superior efficacy in reducing body weight, with all other agents except Mazdutide (MD: -5.31 kg, 95% CI: -9.78 to -0.84, P-score: 0.37, p: 0.0189) achieving reductions of over 8 kg. In patients with type 2 diabetes, all agents reduced HbA_1c_ by over 1%, with Tirzepatide (MD: -1.87%, 95% CI: -2.15 to -1.59, P-score: 0.87, p < 0.0001) and Mazdutide (MD: -1.89%, 95% CI: -2.43 to -1.35, P-score: 0.88, p < 0.0001) showing the greatest effects on glycemic control. For blood pressure management, Tirzepatide significantly reduced systolic blood pressure (MD: -6.69 mmHg, 95% CI: -7.62 to -5.75, P-score: 0.84, p < 0.0001) and diastolic blood pressure (MD: -3.73 mmHg, 95% CI: -4.75 to -2.71, P-score: 0.92, p < 0.0001), with nearly all agents lowering systolic blood pressure by more than 5 mmHg. Non-diabetic participants showed more pronounced improvements in both weight and blood pressure. Safety analysis revealed that Tirzepatide had a favorable safety profile and all agents showed no significant impact on serious adverse events compared to placebo.

**Conclusions:**

Multi-receptor drugs demonstrated substantial therapeutic potential in weight management, glycemic control, and blood pressure regulation in adults with overweight or obesity, with or without diabetes, with a generally favorable safety profile.

**Systematic review registration:**

https://www.crd.york.ac.uk/prospero/, identifier CRD42024554005.

## 1 Introduction

In the 21st century, obesity and type 2 diabetes have emerged as global epidemics. According to the Global Burden of Disease Obesity Collaboration, more than 600 million adults worldwide are affected by obesity, while diabetes impacts approximately 476 million individuals, 463 million of whom have type 2 diabetes (1, 2). These chronic conditions pose serious threats to both physical and mental health, increasing the risk of cardiovascular disease, various cancers, and depression, thereby challenging public health systems globally (3). Additionally, obesity is recognized as an independent and crucial risk factor for the development of type 2 diabetes (4), making weight management essential for both prevention and treatment.

In recent years, significant progress has been made in the treatment of diabetes and obesity with the development of novel therapeutic agents. Glucagon-like peptide-1 receptor agonists (GLP-1RAs) represent a new class of drugs that not only improve glycemic control but also promote weight loss, thereby reducing the risk of cardiovascular diseases (5). Several GLP-1RAs, such as Liraglutide, Semaglutide, and Dulaglutide, have been approved by the U.S. Food and Drug Administration (FDA) for the treatment of overweight or obesity and the management of type 2 diabetes (6–9). Notably, Tirzepatide, a dual glucose-dependent insulinotropic polypeptide receptor (GIPR) and GLP-1R agonist, has demonstrated superior efficacy in both weight loss and glycemic control compared to single GLP-1RAs, such as Liraglutide and Semaglutide (10–13). Furthermore, several RCTs in adults with overweight or obesity have evaluated other multi-receptor drugs, such as Retatrutide, which targets GIPR/Glucagon receptor (GCGR)/GLP-1R, and Mazdutide, which targets GCGR/GLP-1R, showing promising results in both glycemic management and weight reduction (14–17). These findings suggest that targeting GIPR and/or GCGR alongside GLP-1R may represent a more effective therapeutic strategy, potentially yielding superior clinical outcomes compared to single GLP-1RAs.

The development of multi-receptor drugs based on GLP-1R is a rapidly advancing field, with several new drugs, including Survodutide, Efinopegdutide, AMG133, and Retatrutide, showing potential clinical benefits. Although there has been comprehensive comparison among various GLP-1RAs (12), there remains a lack of comprehensive comparisons between different multi-receptor drugs, limiting the availability of sufficient and timely evidence for clinicians, patients, and researchers. Therefore, we conducted a systematic review and network meta-analysis to assess the safety and efficacy of multi-receptor drugs in patients with overweight or obesity, with or without type 2 diabetes. Our study incorporates the most up-to-date and comprehensive RCTs involving multi-receptor drugs targeting GLP-1R, including Survodutide, Mazdutide, Efinopegdutide, Retatrutide, Tirzepatide, and other novel drugs.

## 2 Methods

The protocol for this systematic review and network meta-analysis was registered on PROSPERO (CRD42024554005). This study was conducted in accordance with the Preferred Reporting Items for Systematic Reviews and Network Meta-Analyses (PRISMA-NMA) guidelines.

### 2.1 Search strategy

A comprehensive search was conducted across PubMed, Embase, Web of Science, the Cochrane Central Register of Controlled Trials (CENTRAL), CNKI, and WanFang databases from inception to May 12, 2024. The search strategy combined Medical Subject Headings (MeSH) terms and free-text keywords to identify RCTs involving multi-receptor agonists targeting GLP-1R. No language restrictions were applied. Detailed search strategies are provided in the **Supplementary Materials** (Appendix 1).

### 2.2 Eligibility criteria

Eligible RCTs included patients with overweight or obesity, with or without type 2 diabetes. Participants in the intervention group were treated with the following multi-receptor drugs: Retatrutide, Tirzepatide, Survodutide, Mazdutide, Efinopegdutide, and AMG 133. The control group received either a different multi-receptor drug or a placebo. Studies with a treatment duration of less than 12 weeks or those involving prematurely terminated interventions were excluded. Duplicated studies, conference abstracts, and publications lacking relevant outcomes were also excluded. Two independent reviewers screened the titles and abstracts to exclude irrelevant studies, followed by full-text review of potentially eligible articles based on predefined inclusion and exclusion criteria. Any disagreements in study selection were resolved through discussion with a third reviewer.

### 2.3 Data extraction

Two reviewers independently extracted data from the included studies using a pre-designed form. Extracted information included basic study details (first author, year of publication, clinicaltrials.gov registration number, and treatment duration) and baseline characteristics of the population (age, gender, sample size, and intervention details such as drug name and dosage). To comprehensively assess efficacy and safety, the following outcomes were considered: efficacy (changes from baseline in HbA_1c_, FPG, body weight, the proportion of participants achieving a weight loss of more than 5%, body mass index (BMI), waist circumference, systolic and diastolic blood pressure) and safety (adverse events and serious adverse events). Any discrepancies in data extraction between the two reviewers were resolved through review and evaluation by a third investigator.

### 2.4 Risk of bias assessment

Two reviewers independently assessed the risk of bias in the included trials using the Cochrane Risk of Bias tool for randomized trials. Any discrepancies were resolved through discussion with a third reviewer to reach a consensus. The studies were categorized as having low, some concerns, or high risk of bias. A comparison-adjusted funnel plot was used to assess publication bias, and Egger’s test was performed to quantitatively evaluate the symmetry of the funnel plot (18).

### 2.5 Statistical analysis

A random-effects model was employed for the network meta-analysis under the frequentist framework, with statistical analyses performed using R version 4.3.2 and the ‘netmeta’ package. Continuous outcomes, including HbA_1c_, FPG, body weight, BMI, waist circumference, diastolic blood pressure, and systolic blood pressure, were evaluated using mean differences (MDs) with 95% confidence intervals (CIs). For categorical outcomes, odds ratios (ORs) were calculated for participants achieving a weight loss of more than 5%, adverse events, and serious adverse events, with both efficacy and safety outcomes reported alongside 95% CIs. Secondary analyses were conducted to assess changes in body weight, and systolic and diastolic blood pressure between diabetic and non-diabetic populations. The P-score method was applied to rank the effectiveness and safety of the interventions (19).

### 2.6 Assessment of confidence in findings

We assessed the effect estimates for the primary outcomes using CINeMA framework and methodology (20, 21). This evaluation encompassed six domains: within-study bias, across-study bias, indirectness, imprecision, heterogeneity, and incoherence. Each domain was rated on a three-level scale: no concerns, some concerns, or major concerns. If a domain was rated as “serious,” the quality of evidence was downgraded by one level; if rated as “very serious,” it was downgraded by two levels. The ratings across all domains were then synthesized to provide an overall confidence rating, categorized as low, moderate, or high.

## 3 Results

**Figure 1:**
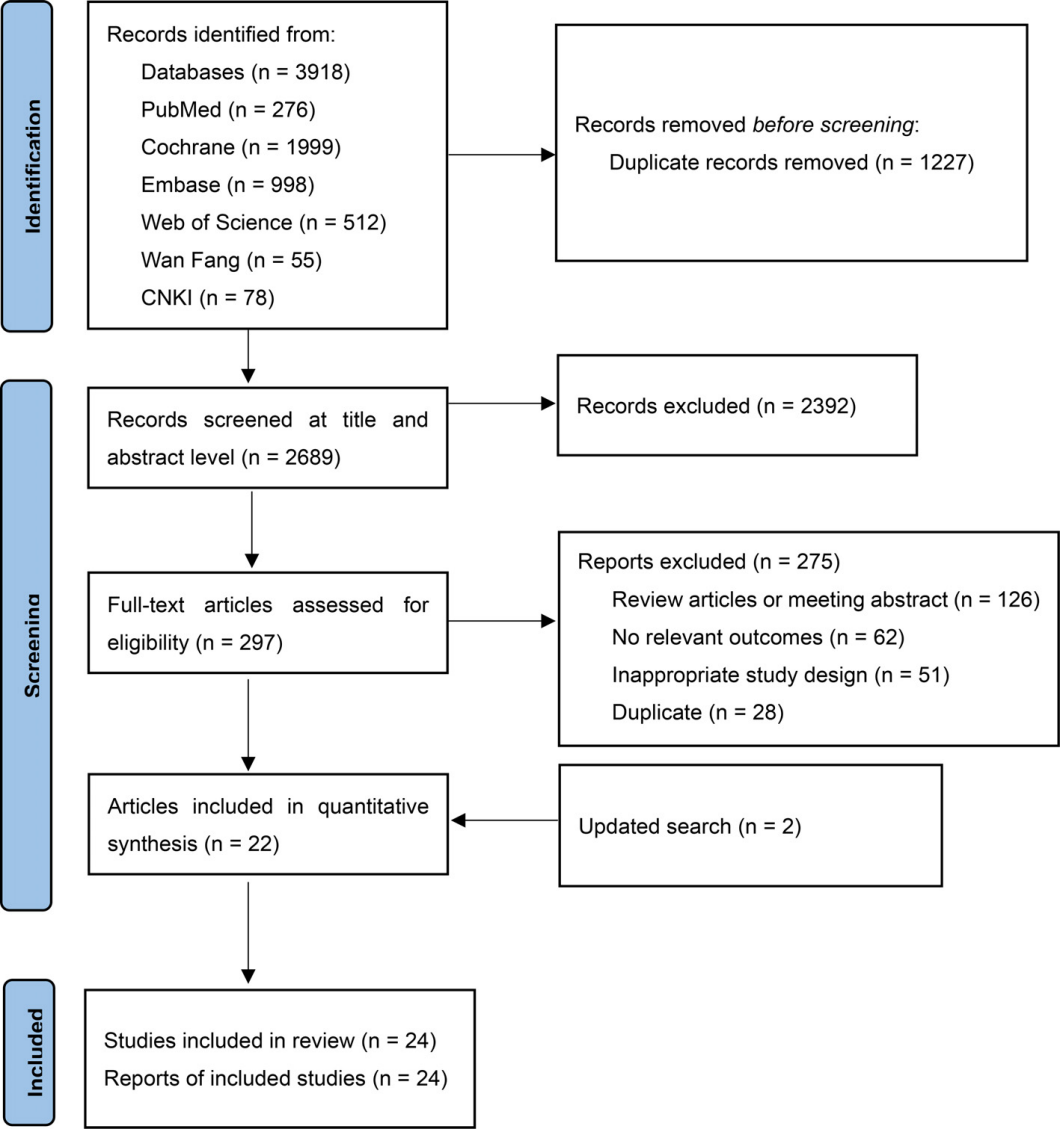
PRISMA flow diagram of the study selection process.

Following our predefined search strategy, a total of 3,918 records were initially identified. After removing 1,227 duplicates and screening titles and abstracts to exclude irrelevant studies, full-text assessments were subsequently conducted for 297 articles (**Figure 1**). Based on the inclusion criteria, there were 24 RCTs involving 9165 participants. Of these, 11 RCTs were conducted in individuals with overweight or obesity and type 2 diabetes (**Table 1**), and the remaining 13 RCTs involved individuals with overweight or obesity but without diabetes (**Table 2**). The sample sizes of the included RCTs ranged from 24 to 2,539 adults, with intervention durations spanning 12 to 72 weeks. A detailed summary of the characteristics of these 24 studies and their participants is provided in **Supplementary Table S12**.

**Table 1:** Study of patients with type 2 diabetes.

Study	Trial registration no.	Study duration	Number of participants	Treatments	
Matthias2024	NCT04153929	16 weeks	361	Placebo: 59	Survodutide: 302
Heise2022	NCT03951753	28 weeks	73	Placebo:28	Tirzepatide: 45
Zhang2024	NCT04965506	20 weeks	200	Placebo:51	Mazdutide:149
Jiang2022	NCT04466904	12 weeks	42	Placebo:12	Mazdutide: 30
Di Prospero2021	NCT03586830	12 weeks	195	Placebo: 49	Efinopegdutide: 146
Dahl2022	NCT04039503	40 weeks	475	Placebo: 120	Tirzepatide:355
Urva2022	NCT04143802	12 weeks	67	Placebo: 15	Retatrutide: 52
Rosenstock2023	NCT04867785	36 weeks	235	Placebo:45	Retatrutide: 190
Rosenstock2021	NCT03954834	40 weeks	478	Placebo:115	Tirzepatide:363
Frias2018	NCT03131687	26 weeks	263	Placebo:51	Tirzepatide: 212
Garvey2023	NCT04657003	72 weeks	938	Placebo:315	Tirzepatide: 623

**Table 2:** Study of patients without type 2 diabetes.

Study	Trial registration no.	Study duration	Number of participants	Treatments	
Wadden2023	NCT04657016	72 weeks	579	Placebo: 292	Tirzepatide: 287
Aronne2024	NCT04660643	52 weeks	770	Placebo: 335	Tirzepatide: 335
Alba2021	NCT03486392	26 weeks	355	Placebo: 60	Efinopegdutide:295
Jastreboff2023	NCT04881760	48 weeks	338	Placebo: 70	Retatrutide: 268
Arun2024	NCT04771273	48 weeks	293	Placebo: 74	Survodutide: 219
Zhao2023	NCT05024032	52 weeks	210	Placebo:69	Tirzepatide: 141
Yazawa2023	NCT04384081	16 weeks	36	Placebo:9	Survodutide:27
Ji2023	NCT04904913	24 weeks	248	Placebo:62	Mazdutide:186
Jastreboff2022	NCT04184622	72 weeks	2539	Placebo:643	Tirzepatide: 1896
Véniant2024	NCT04478708	30 weeks	26	Placebo:6	AMG133:20
Roux2024	NCT04667377	46 weeks	384	Placebo:77	Survodutide:307
Ji2022*	NCT04440345	16 weeks	24	Placebo:8	Mazdutide: 16
Ji2021*	NCT04440345	12 weeks	36	Placebo:12	Mazdutide:24

*: Different dose groups.

**Figure 2:**
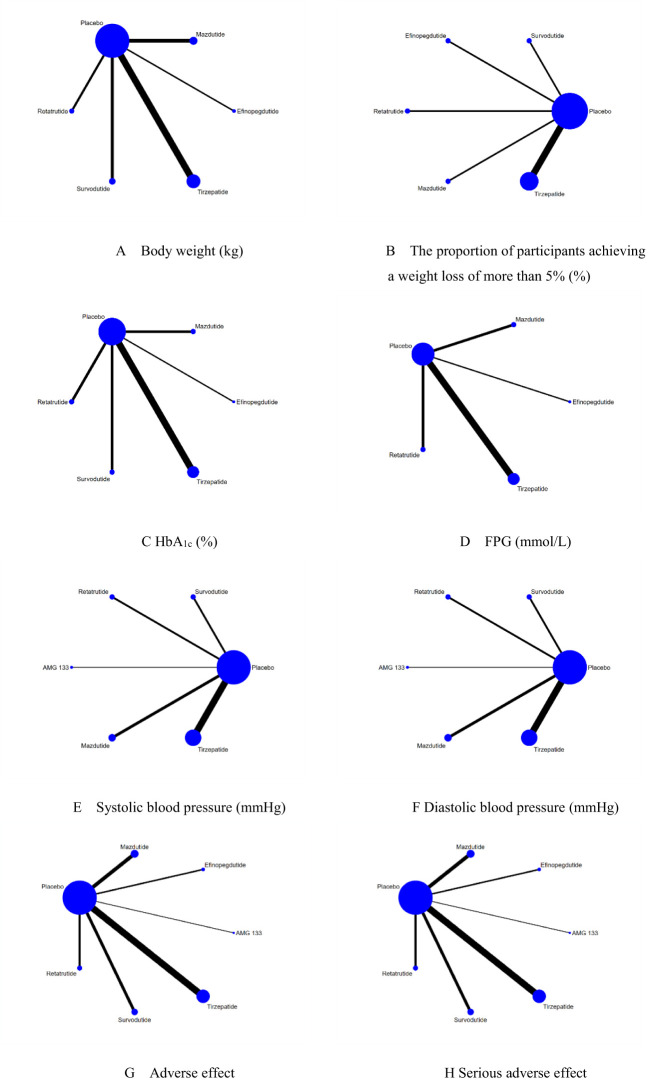
The network diagrams of all eligible comparisons for the primary outcomes of efficacy and safety. **(A)** Body weight (kg); **(B)** The proportion of participants achieving a weight loss of more than 5% (%); **(C)** HbA1c (%); **(D)** FPG (mmol/L); **(E)** Systolic blood pressure (mmHg); **(F)** Diastolic blood pressure (mmHg); **(G)** Adverse effect; **(H)** Serious adverse effect.

Therefore, the network meta-analysis focused on six multi-receptor drugs: one triple GIPR/GCGR/GLP-1R agonist (Retatrutide), one dual GIPR/GLP-1R agonists (Tirzepatide), and three GCGR/GLP-1R agonists (Survodutide, Mazdutide, and Efinopegdutide) and one GLP-1R agonist/GIPR antagonist, bispecific molecule (AMG 133 [maridebart cafraglutide]) (**Figure 2**).

### 3.1 Body weight management

**Figure 3:**
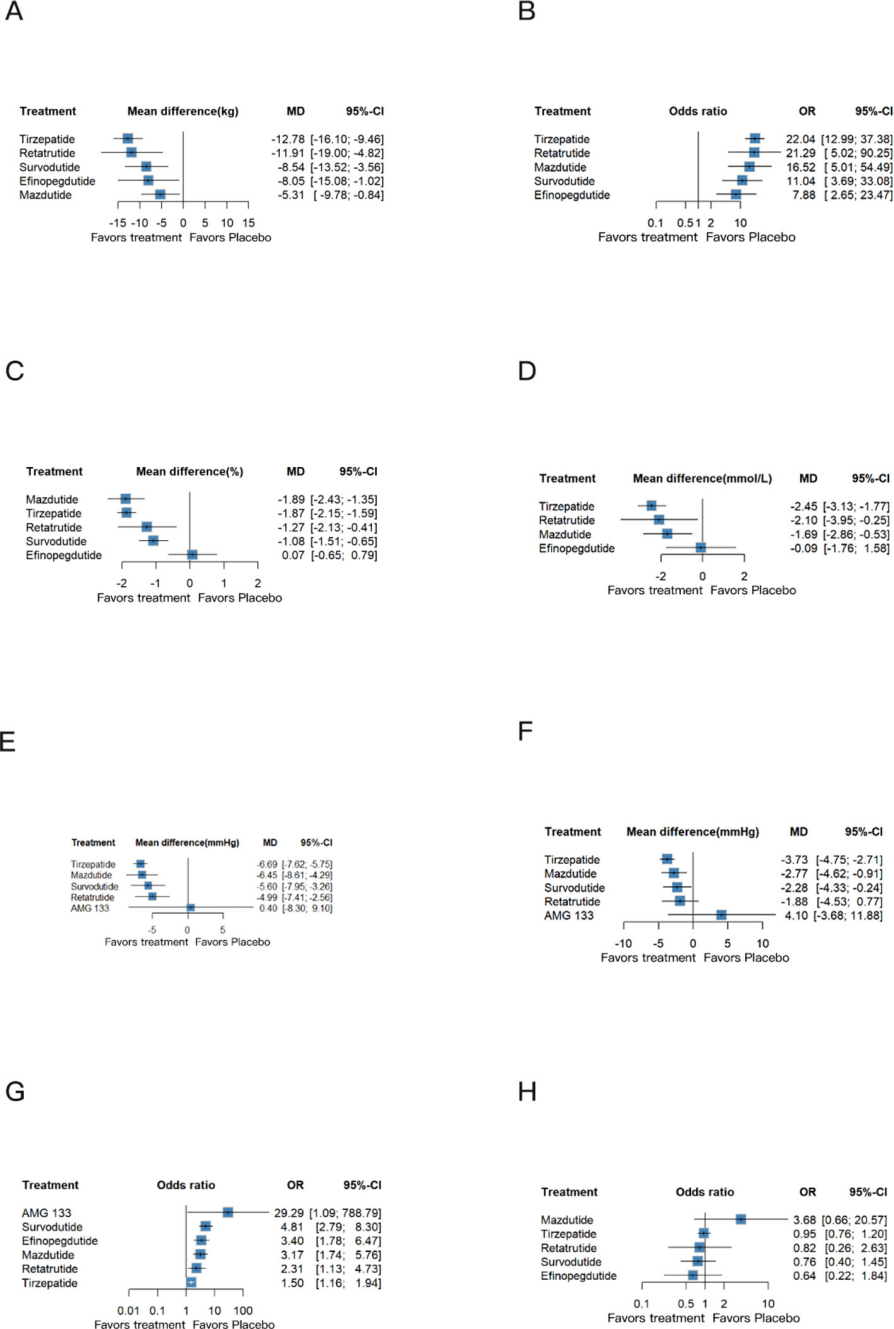
Network meta-analysis results for the primary outcomes of efficacy and safety. **(A)** Body weight (kg); **(B)** The proportion of participants achieving a weight loss of more than 5% (%); **(C)** HbA_1c_ (%); **(D)** FPG (mmol/L); **(E)** Systolic blood pressure (mmHg); **(F)** Diastolic blood pressure (mmHg); **(G)** Adverse effect; **(H)** Serious adverse effect.

To provide a comprehensive overview of weight management outcomes, we included 24 RCTs and analyzed five key indicators: weight loss, BMI, waist circumference, weight reduction in percentage and the proportion of participants achieving a weight loss of more than 5%. Compared to placebo, Tirzepatide demonstrated superior efficacy in reducing body weight (MD: -12.78 kg, 95% CI: -16.10 to -9.46, P-score: 0.89), followed by Retatrutide (MD: -11.91 kg, 95% CI: -19.00 to -4.82, P-score: 0.80), Survodutide (MD: -8.54 kg, 95% CI: -13.52 to -3.56, P-score: 0.59), and Efinopegdutide (MD: -8.05 kg, 95% CI: -15.08 to -1.02, P-score: 0.56). All of these treatments showed significant weight loss effects (**Figure 3A**). Regarding BMI, Tirzepatide demonstrated a remarkable improvement effect (MD: -3.68 kg/m^2^, 95% CI: -6.77 to -0.58, P-score: 0.61) (**Supplementary Figure S3.10**). However, no statistically significant difference was observed in the percentage of weight loss compared to placebo (**Supplementary Figure S3.11**).

Similarly, the reduction in waist circumference followed a trend consistent with the weight loss results. Tirzepatide showed the greatest reduction (MD: -10.86 cm, 95% CI: -13.24 to -8.48, P-score: 0.92), followed by Retatrutide (MD: -9.01 cm, 95% CI: -13.60 to -4.42, P-score: 0.74) and Survodutide (MD: -7.08 cm, 95% CI: -10.12 to -4.05, P-score: 0.57) (**Supplementary Figure S3.2**).Finally, we compared the proportion of participants achieving a weight loss of more than 5%. Tirzepatide was associated with the highest proportion (OR: 22.04, 95% CI: 12.99 to 37.38, P-score: 0.80), closely followed by Retatrutide (OR: 21.29, 95% CI: 5.02 to 90.25, P-score: 0.74), Mazdutide (OR: 16.52, 95% CI: 5.01 to 54.49, P-score: 0.65), and Survodutide (OR: 11.04, 95% CI: 3.69 to 33.08, P-score: 0.47) (**Figure 3B**).

### 3.2 Glycemic regulation

For glycemic control outcomes, the analysis was focused on patients with diabetes. HbA_1c_ levels, reported as percentages, were assessed in 11 RCTs involving 5 distinct agents. FPG, measured in mmol/L, was evaluated in 10 RCTs across four agents. The network meta-analysis indicated that Mazdutide (MD: -1.89%, 95% CI: -2.43 to -1.35, P-score: 0.88) and Tirzepatide (MD: -1.87%, 95% CI: -2.15 to -1.59, P-score: 0.87) demonstrated nearly identical efficacy in significantly reducing HbA_1c_ levels (**Figure 3C**). Retatrutide (MD: -1.27%, 95% CI: -2.13 to -0.41, P-score: 0.57) showed greater efficacy compared to Survodutide (MD: -1.08%, 95% CI: -1.51 to -0.65, P-score: 0.47) in lowering HbA_1c_.

The results for FPG revealed notable differences. Tirzepatide (MD: -2.45 mmol/L, 95% CI: -3.13 to -1.77, P-score: 0.87) achieved the most significant reduction in FPG, followed by Retatrutide (MD: -2.10 mmol/L, 95% CI: -3.95 to -0.25, P-score: 0.73), which demonstrated greater efficacy than Mazdutide (MD: -1.69 mmol/L, 95% CI: -2.86 to -0.53, P-score: 0.61) (**Figure 3D**). Additionally, Efinopegdutide had a neutral impact on both HbA_1c_ and FPG levels (**Figures 3C, D**).

### 3.3 Blood pressure control

Given the limitations of available RCTs, our analysis of blood pressure control included data from 15 studies involving 5 multi-receptor drugs. The network meta-analysis showed that, compared to the placebo, both Tirzepatide (MD: -6.69 mmHg, 95% CI: -7.62 to -5.75, P-score: 0.84) and Mazdutide (MD: -6.45 mmHg, 95% CI: -8.61 to -4.29, P-score: 0.77) exhibited notable and comparable reductions in systolic blood pressure (**Figure 3E**). Survodutide (MD: -5.60 mmHg, 95% CI: -7.95 to -3.26, P-score: 0.60) also demonstrated a significant reduction, surpassing Retatrutide (MD: -4.99 mmHg, 95% CI: -7.41 to -2.56, P-score: 0.53), while AMG133 had a non-significant effect compared to placebo.

The effects on diastolic blood pressure were similar to systolic blood pressure. Tirzepatide (MD: -3.73 mm Hg, 95% CI: -4.75 to -2.71, P-score: 0.92) achieved the most substantial reduction, followed by Mazdutide (MD: -2.77 mm Hg, 95% CI: -4.62 to -0.91, P-score: 0.70) and Survodutide (MD: -2.28 mm Hg, 95% CI: -4.33 to -0.24, P-score: 0.53) (**Figure 3F**).

### 3.4 Adverse events

The network meta-analysis revealed that Tirzepatide (OR: 1.50, 95% CI: 1.16 to 1.94, P-score: 0.80) had an unfavorable safety profile compared to placebo, with significant increase in the incidence of adverse events (**Figure 3G**). Retatrutide ranked second (OR: 2.31, 95% CI: 1.13 to 4.73, P-score: 0.59). Besides, other agents significantly increased the risk of adverse events, particularly AMG133 (OR: 29.29, 95% CI 1.09 to 788.79, P-score: 0.08), which showed the highest risk. Survodutide (OR: 4.81, 95% CI: 2.79 to 8.30, P-score: 0.21), Mazdutide (OR: 3.17, 95% CI 1.74 to 5.76, P-score: 0.43), and Efinopegdutide (OR: 3.40, 95% CI: 1.78 to 6.47, P-score: 0.39) also displayed similar adverse event risk profiles. Serious adverse events, as defined by included studies, encompass events that are life-threatening, result in death, require hospitalization or prolong an existing hospitalization, cause persistent disability or incapacity, or involve congenital anomalies or birth defects. The network meta-analysis results indicated that these drugs did not show a notable difference in the incidence of serious adverse events compared to placebo (**Figure 3H**). Detailed information on serious adverse events is provided in Appendix 13.

### 3.5 Subgroup analysis

#### 3.5.1 Subgroup analyses of patients with diabetes and without diabetes

We conducted a subgroup analysis focusing on weight changes and blood pressure alterations in populations with overweight or obesity, with and without type 2 diabetes. The analysis included both weight loss and changes in systolic and diastolic blood pressure.

Among patients with diabetes, compared to placebo, Tirzepatide (MD: -8.77 kg, 95% CI: -11.29 to -6.25, P-score: 0.84) and Survodutide (MD: -7.91 kg, 95% CI: -11.80 to -4.02, P-score: 0.73) demonstrated superior weight reduction compared to Efinopegdutide (MD: -6.56 kg, 95% CI: -12.09 to -1.03, P-score: 0.60) and Retatrutide (MD: -6.18 kg, 95% CI: -11.88 to -0.48, P-score: 0.56) (**Supplementary Figure S8.1**). Similarly, a significantly higher proportion of patients achieved more than 5% weight loss with Tirzepatide (OR: 14.57, 95% CI: 9.66 to 21.98, P-score: 0.83) and Survodutide (OR: 13.33, 95% CI: 4.18 to 42.48, P-score: 0.74) compared to Retatrutide (OR: 10.14, 95% CI: 4.12 to 24.93, P-score: 0.61) and Efinopegdutide (OR: 7.38, 95% CI: 2.62 to 20.78, P-score: 0.46) (**Supplementary Figure S8.3**). Among patients with type 2 diabetes, no statistically significant difference was observed in the percentage of weight loss compared to placebo (**Supplementary Figure S8.9**). However, in patients without diabetes, Tirzepatide (MD: -16.83%, 95% CI: -20.94 to -12.73, P-score: 0.89) demonstrated the greatest weight reduction, followed by Retatrutide (MD: -15.95%, 95% CI: -23.06 to -8.84, P-score: 0.82), which also exhibited a significant weight loss effect (**Supplementary Figure S8.10**).

In patients with overweight or obesity, without type 2 diabetes, Tirzepatide (MD: -17.75 kg, 95% CI: -20.88 to -14.61, P-score: 0.90) and Retatrutide (MD: -17.69 kg, 95% CI: -24.11 to -11.27, P-score: 0.89) were associated with greater weight reduction compared to Efinopegdutide (MD: -9.56 kg, 95% CI: -15.87 to -3.25, P-score: 0.46) and Survodutide (MD: -9.14 kg, 95% CI: -13.66 to -4.62, P-score: 0.44) (**Supplementary Figure S8.2**). For the proportion of patients achieving at least a 5% reduction in body weight, Mazdutide (OR: 54.99, 95% CI: 5.27 to 573.24, P-score: 0.83) showed the most substantial effect, followed by Tirzepatide (OR: 27.48, 95% CI: 9.62 to 78.51, P-score: 0.71) and Retatrutide (OR: 21.79, 95% CI: 2.57 to 176.18, P-score: 0.62) (**Supplementary Figure S8.4**).

Among patients with overweight or obesity and with type 2 diabetes, various treatments showed similar effects in reducing systolic blood pressure. Survodutide (MD: -5.81 mmHg, 95% CI: -10.27 to -1.35, P-score: 0.66), Tirzepatide (MD: -5.80 mmHg, 95% CI: -8.53 to -3.06, P-score: 0.67), and Mazdutide (MD: -5.79 mmHg, 95% CI: -10.38 to -1.20, P-score: 0.66) exhibited comparable outcomes (**Supplementary Figure S8.5**). Mazdutide (MD: -2.68 mmHg, 95% CI: -4.95 to -0.41, P-score: 0.72) showed the most pronounced reductions in diastolic blood pressure, followed by Tirzepatide (MD: -2.45 mmHg, 95% CI: -3.61 to -1.28, P-score: 0.67) (**Supplementary Figure S8.6**).

In patients with overweight or obesity, without type 2 diabetes, Tirzepatide (MD: -6.99 mmHg, 95% CI: -8.38 to -5.61, P-score: 0.82) was still the most effective agent for lowering systolic blood pressure, followed closely by Mazdutide (MD: -6.88 mmHg, 95% CI: -9.87 to -3.89, P-score: 0.78). Survodutide (MD: -5.43 mmHg, 95% CI: -9.00 to -1.86, P-score: 0.58) and Retatrutide (MD: -5.19 mmHg, 95% CI: -8.48 to -1.90, P-score: 0.55) were the next most effective agents (**Supplementary Figure S8.7**). In terms of diastolic blood pressure reduction, Tirzepatide (MD: -4.32 mmHg, 95% CI: -5.56 to -3.08, P-score: 0.94) was significantly more effective than Mazdutide (MD: -2.81 mmHg, 95% CI: -5.17 to -0.46, P-score: 0.68) (**Supplementary Figure S8.8**).

#### 3.5.2 Subgroup analyses of each multi-receptor drug with multiple doses

We conducted subgroup analyses to evaluate the efficacy of six drugs at various doses. Different multi-receptor drugs demonstrated varying degrees of superiority over placebo across a range of treatment outcomes. Retatrutide, administered at 12 mg and 8 mg (both fast and slow formulations), exhibited the greatest weight loss, outperforming Tirzepatide (15 mg). Survodutide (3.6 mg, 4.8 mg, 6.0 mg) at lower doses also showed comparable weight loss to Tirzepatide (15 mg) (**Supplementary Figure S11.3**). For BMI outcomes, Retatrutide (12 mg, 8 mg fast, 8 mg slow, 4 mg fast) and Survodutide (6.0 mg, 4.8 mg, 3.6 mg) were the most effective, followed by the highest dose of Tirzepatide (15 mg) (**Supplementary Figure S11.10**). In terms of waist circumference, Retatrutide at 8 mg (both fast and slow formulations) and 12 mg produced the most significant reduction, followed by Tirzepatide (15 mg) and Survodutide (1.8 mg biw) (**Supplementary Figure S11.5**). Regarding glycemic control, Tirzepatide (15 mg, 10 mg) was most effective in reducing HbA_1c_ levels (**Supplementary Figure S11.1**). For FPG, Retatrutide (1.5 mg, 8 mg slow) and Mazdutide (6 mg) achieved the most notable reductions (**Supplementary Figure S11.2**). AMG133 exhibited the greatest reduction in blood pressure effects at the 280 mg dose. Retatrutide (8 mg) and Mazdutide (6 mg) also significantly reduced systolic blood pressure (**Supplementary Figure S11.6**, **Supplementary Figure S11.7**).

Significant increase in adverse events was observed with Tirzepatide at most doses (5 mg, 10 mg, 15mg). Retatrutide at the 4 mg (slow) dose demonstrated a favorable safety profile, whereas Survodutide at lower doses (0.3 mg, 0.9 mg, 1.2 mg, 1.8 mg, 2.7 mg) was associated with a higher incidence of adverse reactions. Most multi-receptor drugs did not significantly increase the risk of serious adverse events compared to placebo (**Supplementary Figure S11.8** and **Supplementary Figure S11.9**).

## 4 Discussion

In this network meta-analysis, we comprehensively evaluated the efficacy and safety of multi-receptor drugs, focusing on six agents: one triple GIPR/GCGR/GLP-1R agonist (Retatrutide), one dual GIPR/GLP-1R agonist (Tirzepatide), and three GCGR/GLP-1R agonists (Survodutide, Mazdutide, and Efinopegdutide) and one GLP-1R agonist/GIPR antagonist, bispecific molecule (AMG 133 [maridebart cafraglutide]). A total of 24 eligible RCTs and 9165 patients were included, assessing outcomes such as weight reduction, glycemic control, blood pressure changes, and safety profiles. Subgroup analyses were performed to explore the efficacy across different dosage regimens. Furthermore, the CINeMA framework was used to assess confidence in the network meta-analysis results.

Overall, all drugs demonstrated substantial reductions in weight (exceeding 5 kg) and HbA_1c_ levels (greater than 1%), indicating promising potential for clinical application. Among the multi-receptor drugs, Tirzepatide and Retatrutide demonstrated the most significant weight loss. Subgroup analyses revealed that Survodutide also produced substantial weight loss, second only to Tirzepatide among patients with type 2 diabetes.

For glycemic control, Mazdutide and Tirzepatide were the most effective in lowering HbA_1c_, displaying nearly equivalent efficacy. In contrast, Efinopegdutide had a minimal impact on glycemic levels. Regarding blood pressure, Tirzepatide and Mazdutide produced the most favorable outcomes. However, AMG133 demonstrated variable dosing effects on blood pressure, with different doses leading to distinct changes in both systolic and diastolic pressure, highlighting the need for further investigation. In terms of safety, Tirzepatide had the most favorable profile, while other drugs showed a notable elevation in the risk of adverse reactions. However, none of the drugs increased the incidence of serious adverse events compared to placebo. Our study showed the overall efficacy of multi-receptor drugs in reducing HbA_1c_, FPG, managing weight, and controlling blood pressure.

Subgroup analyses revealed that weight loss and blood pressure reductions were more pronounced in non-diabetic populations than in patients with type 2 diabetes. This discrepancy could result from differences in metabolic status and insulin resistance in diabetic patients. Non-diabetic individuals typically exhibit less insulin resistance or hyperglycemia, allowing them to derive more direct benefits from the blood pressure improvements associated with weight loss through multi-receptor drugs (2, 22). The close relationship between weight loss and blood pressure reduction suggests that greater weight loss often leads to more significant blood pressure improvements (23). Additionally, dose-response subgroup analyses showed that Tirzepatide exhibits a dose-dependent effect on both weight and HbA_1c_ reductions, while Efinopegdutide also exhibited dose-dependent effects on weight management. In contrast, Retatrutide, Survodutide, and Mazdutide achieved significant clinical outcomes even at lower doses.

Despite these strengths, our study has several limitations. First, although our aim was to conduct direct comparisons between multi-receptor drugs, the limited availability of head-to-head trials among these drugs in existing RCTs precluded such analyses. Consequently, our network meta-analysis focuses on a comprehensive comparison between multi-receptor drugs and placebo, in accordance with the predefined inclusion criteria. Second, the number of RCTs for certain drugs was limited, which may affect confidence in some findings. Gender differences may also influence the safety and efficacy of multi-receptor drugs, as women tend to experience greater reductions in blood glucose and body weight (24). Moreover, racial differences may play a role in treatment outcomes. For instance, East Asian patients with type 2 diabetes typically have lower obesity rates and reduced insulin resistance compared to Western populations. However, the limited number of studies involving Asian populations precluded subgroup analyses based on gender and race (25)

Most meta-analyses focus on GLP-1R agonists, lacking detailed meta-analyses comparing between multi-receptor drugs (5, 13, 26–29). Our study addresses this gap by conducting detailed analysis and subgroup analyses in adults with overweight or obesity, with or without type 2 diabetes, and across all available dosages of multi-receptor drugs. This analysis ranks the efficacy and safety of the drugs, providing valuable insights for future drug development and clinical decision-making.

The therapeutic potential of multi-receptor drugs extends beyond their established roles in glycemic control and weight management. Research is expanding their use into other therapeutic areas, including non-alcoholic steatohepatitis (NASH) and non-alcoholic fatty liver disease (NAFLD) (30, 31), in which these agents have been shown to reduce liver fat content. They have also been explored for treating conditions such as obstructive sleep apnea (32) and for providing cardiovascular benefits, including lowering blood pressure and reducing the risk of cardiovascular events (33, 34). Preliminary studies suggest that multi-receptor drugs might exhibit neuroprotective effects in conditions such as Alzheimer’s disease, potentially reducing neuroinflammation and slowing disease progression (35). The demonstrated multifaceted efficacy of these agents, particularly in glycemic control, weight management, and cardiovascular protection, positions them as promising candidates for a broad range of clinical applications. Further investigation into their role in future treatment paradigms is warranted, as they may significantly enhance current medical approaches across a variety of conditions.

## 5 Conclusion

Our network meta-analysis of 24 RCTs reveals that multi-receptor drugs significantly reduce body weight and achieve clinically meaningful reductions in blood pressure compared to placebo, across populations with overweight or obesity, both with and without type 2 diabetes. These agents also notably lower HbA_1c_ and FPG in patients with type 2 diabetes. Multi-receptor drugs demonstrate superior efficacy in non-diabetic individuals, achieving greater reductions in both systolic and diastolic blood pressure and more pronounced weight loss compared to those with type 2 diabetes. While most agents exhibit generally favorable safety profiles, some drugs are potentially linked to an increased risk of adverse reactions. Overall, this analysis highlights the extensive therapeutic potential of multi-receptor drugs, especially in non-diabetic populations, offering promising benefits for obesity management, glycemic control, and blood pressure regulation.

## Data Availability

The raw data supporting the conclusions of this article will be made available by the authors upon reasonable request.
