# Supplementary material for: Comparative efficacy of incretin drugs on glycemic control, body weight, and blood pressure in adults with overweight or obesity and with/without type 2 diabetes: a systematic review and network meta-analysis
Source: Front Endocrinol (Lausanne). 2025 Feb 4;16:1513641. doi: 10.3389/fendo.2025.1513641 (PMC11832366; doi:10.3389/fendo.2025.1513641)
Supplement: Supplementary file 1 [file DataSheet1.pdf]

Comparative efficacy of multi-receptor drugs on glycemic control, body weight, and blood pressure in overweight or obese adults with/without type 2 diabetes: a systematic review and network meta-analysis

Supplementary Appendix

Table of contents

Comparative efficacy of multi-receptor drugs on glycemic control, body weight, and blood pressure in overweight or obese adults with/without type 2 diabetes: a systematic review and network meta-analysis 1
Appendix 1: Search strategy 2
Appendix 2: Risk of bias of randomized clinical trials 8
Appendix 3: Network maps and forest plots of outcomes 9
Appendix 4: Treatment ranking (P-scores) for the effects of various multi-receptor drugs 20
Appendix 5: League Table of Summary Estimates for Multi-receptor Drugs Derived from Network Meta-analysis of 24 Trials 21
Appendix 6: CINeMA Assessment 31
Appendix 7: Funnel plots 38
Appendix 8: Subgroup analysis of multi-receptor drugs on patients with or without T2D 44
Appendix 9: Treatment ranking (P-scores) for the effects of Subgroup analysis of multi-receptor drugs on patients with or without T2D 54
Appendix 10: League Table of Summary Estimates for Subgroup analysis of multi-receptor drugs on patients with or without T2D 55
Appendix 11: Comprehensive comparisons of different multi-receptor drugs at various doses 64
Appendix 12: Study details and participant baseline characteristics of included arms in RCT 74
Appendix 13: Definition of serious Adverse Events (SAEs). 80

Appendix 1: Search strategy

Table S1. Search strategy of PubMed

1	"Glucagon-Like Peptide 1"[Mesh]
2	(((Glucagon-Like Peptide-1[Title/Abstract]) OR (Glucagon Like Peptide 1[Title/Abstract])) OR (GLP-1[Title/Abstract])) OR (GLP 1[Title/Abstract])
3	"Receptors, Glucagon"[Mesh]
4	(((Glucagon Receptor[Title/Abstract]) OR (Receptor, Glucagon[Title/Abstract])) OR (Glucagon Receptors[Title/Abstract])) OR (GCGR[Title/Abstract])
5	"gastric inhibitory polypeptide receptor" [Supplementary Concept]
6	((((receptor, gastric inhibitory polypeptide[Title/Abstract]) OR (glucose-dependent insulinotropic polypeptide receptor[Title/Abstract])) OR (gastric inhibitory polypeptide receptors[Title/Abstract])) OR (GIP receptor[Title/Abstract])) OR (GIPR[Title/Abstract])
7	("Glucagon-Like Peptide 1"[Mesh]) OR ((((Glucagon-Like Peptide-1[Title/Abstract]) OR (Glucagon Like Peptide 1[Title/Abstract])) OR (GLP-1[Title/Abstract])) OR (GLP 1[Title/Abstract]))
8	("Receptors, Glucagon"[Mesh]) OR ((((Glucagon Receptor[Title/Abstract]) OR (Receptor, Glucagon[Title/Abstract])) OR (Glucagon Receptors[Title/Abstract])) OR (GCGR[Title/Abstract]))
9	("gastric inhibitory polypeptide receptor" [Supplementary Concept]) OR (((((receptor, gastric inhibitory polypeptide[Title/Abstract]) OR (glucose-dependent insulinotropic polypeptide receptor[Title/Abstract])) OR (gastric inhibitory polypeptide receptors[Title/Abstract])) OR (GIP receptor[Title/Abstract])) OR (GIPR[Title/Abstract]))
10	((("gastric inhibitory polypeptide receptor" [Supplementary Concept]) OR (((((receptor, gastric inhibitory polypeptide[Title/Abstract]) OR (glucose-dependent insulinotropic polypeptide receptor[Title/Abstract])) OR (gastric inhibitory polypeptide receptors[Title/Abstract])) OR (GIP receptor[Title/Abstract])) OR (GIPR[Title/Abstract]))) OR (("Receptors, Glucagon"[Mesh]) OR ((((Glucagon Receptor[Title/Abstract]) OR (Receptor, Glucagon[Title/Abstract])) OR (Glucagon Receptors[Title/Abstract])) OR (GCGR[Title/Abstract])))) AND (("Glucagon-Like Peptide 1"[Mesh]) OR ((((Glucagon-Like Peptide-1[Title/Abstract]) OR (Glucagon Like Peptide 1[Title/Abstract])) OR (GLP-1[Title/Abstract])) OR (GLP 1[Title/Abstract])))
11	((GLP/GCGR[Title/Abstract]) OR (GLP/GIP[Title/Abstract])) OR (GLP/GCGR/GIP[Title/Abstract])
12	(((GLP/GCGR[Title/Abstract]) OR (GLP/GIP[Title/Abstract])) OR (GLP/GCGR/GIP[Title/Abstract])) OR (((("gastric inhibitory polypeptide receptor" [Supplementary Concept]) OR (((((receptor, gastric inhibitory polypeptide[Title/Abstract]) OR (glucose-dependent insulinotropic polypeptide receptor[Title/Abstract])) OR (gastric inhibitory polypeptide receptors[Title/Abstract])) OR (GIP receptor[Title/Abstract])) OR (GIPR[Title/Abstract]))) OR (("Receptors, Glucagon"[Mesh]) OR ((((Glucagon Receptor[Title/Abstract]) OR (Receptor, Glucagon[Title/Abstract])) OR (Glucagon Receptors[Title/Abstract])) OR (GCGR[Title/Abstract])))) AND (("Glucagon-Like Peptide 1"[Mesh]) OR ((((Glucagon-Like Peptide-1[Title/Abstract]) OR (Glucagon Like Peptide 1[Title/Abstract])) OR (GLP-1[Title/Abstract])) OR (GLP 1[Title/Abstract]))))
13	(((((((((((survodutide[Title/Abstract]) OR (BI-456906[Title/Abstract])) OR (AMG133[Title/Abstract])) OR (maridebart cafraglutide[Title/Abstract])) OR (CT-

868[Title/Abstract])) OR (LY2944876[Title/Abstract])) OR (Pegapamodutide[Title/Abstract])) OR (MEDI0382[Title/Abstract])) OR (Cotadutide[Title/Abstract])) OR ((Pemvidutide[Title/Abstract]) OR (ALT-801[Title/Abstract]))) OR (((retatrutide[Title/Abstract]) OR (LY3437943[Title/Abstract])) OR ("retatrutide" [Supplementary Concept]))) OR ((((mazdutide[Title/Abstract]) OR (IBI362[Title/Abstract])) OR (LY3305677[Title/Abstract])) OR ("mazdutide" [Supplementary Concept]))) OR ((((zepbound[Title/Abstract]) OR (LY3298176[Title/Abstract])) OR (tirzepatide[Title/Abstract])) OR ("tirzepatide" [Supplementary Concept]))

14 ((randomized controlled trial[Publication Type]) OR (randomized[Title/Abstract])) OR (placebo[Title/Abstract])

15 ((((GLP/GCGR[Title/Abstract]) OR (GLP/GIP[Title/Abstract])) OR (GLP/GCGR/GIP[Title/Abstract])) OR (((("gastric inhibitory polypeptide receptor" [Supplementary Concept]) OR (((((receptor, gastric inhibitory polypeptide[Title/Abstract]) OR (glucose-dependent insulinotropic polypeptide receptor[Title/Abstract])) OR (gastric inhibitory polypeptide receptors[Title/Abstract])) OR (GIP receptor[Title/Abstract])) OR (GIPR[Title/Abstract]))) OR (("Receptors, Glucagon"[Mesh]) OR ((((Glucagon Receptor[Title/Abstract]) OR (Receptor, Glucagon[Title/Abstract])) OR (Glucagon Receptors[Title/Abstract])) OR (GCGR[Title/Abstract])))) AND (("Glucagon-Like Peptide 1"[Mesh]) OR ((((Glucagon-Like Peptide-1[Title/Abstract]) OR (Glucagon Like Peptide 1[Title/Abstract])) OR (GLP-1[Title/Abstract])) OR (GLP 1[Title/Abstract]))))) OR ((((((((((((survodutide[Title/Abstract]) OR (BI-456906[Title/Abstract])) OR (AMG133[Title/Abstract])) OR (maridebart cafraglutide[Title/Abstract])) OR (CT-868[Title/Abstract])) OR (LY2944876[Title/Abstract])) OR (Pegapamodutide[Title/Abstract])) OR (MEDI0382[Title/Abstract])) OR (Cotadutide[Title/Abstract])) OR ((Pemvidutide[Title/Abstract]) OR (ALT-801[Title/Abstract]))) OR (((retatrutide[Title/Abstract]) OR (LY3437943[Title/Abstract])) OR ("retatrutide" [Supplementary Concept]))) OR ((((mazdutide[Title/Abstract]) OR (IBI362[Title/Abstract])) OR (LY3305677[Title/Abstract])) OR ("mazdutide" [Supplementary Concept]))) OR ((((zepbound[Title/Abstract]) OR (LY3298176[Title/Abstract])) OR (tirzepatide[Title/Abstract])) OR ("tirzepatide" [Supplementary Concept])))

16 (((((GLP/GCGR[Title/Abstract]) OR (GLP/GIP[Title/Abstract])) OR (GLP/GCGR/GIP[Title/Abstract])) OR (((("gastric inhibitory polypeptide receptor" [Supplementary Concept]) OR (((((receptor, gastric inhibitory polypeptide[Title/Abstract]) OR (glucose-dependent insulinotropic polypeptide receptor[Title/Abstract])) OR (gastric inhibitory polypeptide receptors[Title/Abstract])) OR (GIP receptor[Title/Abstract])) OR (GIPR[Title/Abstract]))) OR (("Receptors, Glucagon"[Mesh]) OR ((((Glucagon Receptor[Title/Abstract]) OR (Receptor, Glucagon[Title/Abstract])) OR (Glucagon Receptors[Title/Abstract])) OR (GCGR[Title/Abstract])))) AND (("Glucagon-Like Peptide 1"[Mesh]) OR ((((Glucagon-Like Peptide-1[Title/Abstract]) OR (Glucagon Like Peptide 1[Title/Abstract])) OR (GLP-1[Title/Abstract])) OR (GLP 1[Title/Abstract]))))) OR ((((((((((((survodutide[Title/Abstract]) OR (BI-456906[Title/Abstract])) OR (AMG133[Title/Abstract])) OR (maridebart cafraglutide[Title/Abstract])) OR (CT-868[Title/Abstract])) OR (LY2944876[Title/Abstract])) OR (Pegapamodutide[Title/Abstract])) OR (MEDI0382[Title/Abstract])) OR (Cotadutide[Title/Abstract])) OR

((Pemvidutide[Title/Abstract]) OR (ALT-801[Title/Abstract]))) OR (((retatrutide[Title/Abstract]) OR (LY3437943[Title/Abstract])) OR ("retatrutide" [Supplementary Concept]))) OR ((((mazdutide[Title/Abstract]) OR (IBI362[Title/Abstract])) OR (LY3305677[Title/Abstract])) OR ("mazdutide" [Supplementary Concept]))) OR ((((zepbound[Title/Abstract]) OR (LY3298176[Title/Abstract])) OR (tirzepatide[Title/Abstract])) OR ("tirzepatide" [Supplementary Concept])))) AND (((randomized controlled trial[Publication Type]) OR (randomized[Title/Abstract])) OR (placebo[Title/Abstract]))

Table S2. Search strategy of Cochrane Central Register of Controlled Trials (CENTRAL)

1	MeSH descriptor: [Glucagon-Like Peptide 1] explode all trees
2	(Glucagon-Like Peptide-1):ti,ab,kw OR (Glucagon Like Peptide-1):ti,ab,kw OR (Glucagon Like Peptide 1):ti,ab,kw OR (GLP-1):ti,ab,kw OR (GLP 1):ti,ab,kw
3	#1 OR #2
4	MeSH descriptor: [Receptors, Glucagon] explode all trees
5	(Glucagon Receptor):ti,ab,kw OR (Receptor, Glucagon):ti,ab,kw OR (Glucagon Receptors):ti,ab,kw OR (GCGR):ti,ab,kw
6	#4 OR #5
7	gastric inhibitory polypeptide receptor):ti,ab,kw OR (receptor, gastric inhibitory polypeptid):ti,ab,kw OR (glucose-dependent insulinotropic polypeptide receptor):ti,ab,kw OR (GIP receptor):ti,ab,kw OR (GIPR):ti,ab,kw
8	#3 AND (#6 OR #7)
9	(tirzepatide):ab,ti,kw OR (zepbound):ab,ti,kw OR (ly3298176):ab,ti,kw OR (mazdutide):ab,ti,kw OR (ibi362):ab,ti,kw OR (ly3305677):ab,ti,kw OR (retatrutide):ab,ti,kw OR (ly3437943):ab,ti,kw OR (pemvidutide):ab,ti,kw OR (alt-801):ab,ti,kw OR (survodutide):ab,ti,kw OR (bi-456906):ab,ti,kw OR (amg133):ab,ti,kw OR (maridebart cafraglutide):ab,ti,kw OR (vk2735):ab,ti,kw OR (ct-868):ab,ti,kw OR (pegapamodutide):ab,ti,kw OR (ly2944876):ab,ti,kw OR (cotadutide):ab,ti,kw OR (medi0382):ab,ti,kw
10	#8 OR #9
11	(randomized control trial):ti,ab,kw OR (randomized):ti,ab,kw OR (placebo):ti,ab,kw
12	#10 AND #11

Table S3. Search strategy of Web of Science

1	TS=(Glucagon-Like Peptide 1 OR Glucagon-Like Peptide-1 OR Glucagon Like Peptide 1 OR GLP-1 OR GLP 1)
2	TS=("Glucagon Receptor*" OR GCGR)
3	TS=(gastric inhibitory polypeptide receptor* OR gastric inhibitory polypeptide OR glucose-dependent insulinotropic polypeptide receptor OR GIP receptor OR GIPR)
4	#3 OR #2
5	#4 AND #1
6	TS=(survodutide OR BI-456906 OR AMG133 OR maridebart cafraglutideOR CT-868 OR LY2944876 OR Pegapamodutide OR MEDI0382 OR Cotadutide OR PemvidutideOR ALT-801 OR retatrutide OR LY3437943 OR retatrutide OR mazdutide OR IBI362 OR LY3305677 OR mazdutide OR zepbound OR LY3298176 OR tirzepatide)
7	#6 OR #5
8	TS=(Random* controlled trial OR random* OR placebo)
9	#8 AND #7

Table S4. Search strategy of Embase

1	'glucagon like peptide 1'/exp OR 'glucagon like peptide 1':ab,ti OR 'glp-1':ab,ti OR 'glp 1':ab,ti OR 'glucagon-like peptide 1':ab,ti OR 'glucagon-like peptide-1':ab,ti
2	'gastric inhibitory polypeptide'/exp OR 'glucose dependent insulinotropic peptide':ab,ti OR 'glucose dependent insulinotropic polypeptide':ab,ti OR 'stomach inhibitory peptide':ab,ti OR 'stomach inhibitory polypeptide':ab,ti OR 'gastric inhibitory polypeptide':ab,ti OR 'gip':ab,ti
3	'glucagon receptor'/exp OR 'glucagon receptor':ab,ti OR 'receptor, glucagon':ab,ti OR 'receptors, glucagon':ab,ti
4	#2 OR #3
5	#1 AND #4
6	'tirzepatide':ab,ti OR 'zepbound':ab,ti OR 'ly3298176':ab,ti OR 'mazdutide':ab,ti OR 'ibi362':ab,ti OR 'ly3305677':ab,ti OR 'retatrutide':ab,ti OR 'ly3437943':ab,ti OR 'pemvidutide':ab,ti OR 'alt-801':ab,ti OR 'survodutide':ab,ti OR 'bi-456906':ab,ti OR 'amg133':ab,ti OR 'maridebart cafraglutide':ab,ti OR 'vk2735':ab,ti OR 'ct-868':ab,ti OR 'pegapamodutide':ab,ti OR 'ly2944876':ab,ti OR 'cotadutide':ab,ti OR 'medi0382':ab,ti
7	#5 OR #6
8	'random':ab,ti OR 'placebo':ab,ti OR 'dobuble-blind':ab,ti
9	#7 AND #8

Appendix 2: Risk of bias of randomized clinical trials

Unique ID	Study ID	Randomization process	Deviations from intended interventions	Missing outcome data	Measurement of the outcome	Selection of the reported result	Over all
Heise2022	NCT03951753	Low	Low	Low	Low	Low	Low
zhang2024	NCT04965506	Low	Low	Low	Low	Low	Low
Yazawa2023	NCT04384081	Low	Low	Low	Low	Low	Low
Ji2023	NCT04904913	Low	Some concerns	Low	Low	Low	Some concerns
Jiang2022	NCT04466904	Low	Some concerns	Low	Low	Low	Some concerns
Jastreboff2022	NCT04184622	Some concerns	Some concerns	Low	Low	Low	Some concerns
Véniant2024	NCT04478708	Some concerns	Low	Low	Low	Some concerns	Some concerns
Roux2024	NCT04667377	Low	Low	Low	Low	Low	Low
Di2021	NCT03586830	Low	Low	Low	Low	Low	Low
Ji2022	NCT04440345	Low	Low	Low	Low	Low	Low
Ji2021	NCT04440345	Low	Low	Low	Low	Low	Low
Dahl2022	NCT04039503	Low	Low	Low	Low	Low	Low
Wadden2024	NCT04657016	Low	Some concerns	Low	Low	Low	Some concerns
Rosenstock2023	NCT04867785	Low	Low	Low	Low	Low	Low
Rosenstock2021	NCT03954834	Low	Low	Low	Low	Low	Low
Frias2018	NCT03131687	Low	Some concerns	Low	Low	Low	Some concerns
Garvey2023	NCT04657003	Low	Low	Low	Low	Low	Low
Aronne2024	NCT04660643	Low	Low	Low	Low	Low	Low
Jastreboff2023	NCT04881760	Low	Some concerns		Low	Low	Some concerns
Matthias2023	NCT04153929	Low	Low	High	Low	Some concerns	High
Alba2020	NCT03486392	Low	High	Low	Low	Low	High
Arun2024	NCT04771273	Low	Low	Low	Low	Low	Low
Zhao2024	NCT05024032	Low	Low	Low	Low	Low	Low
Urva2022	NCT04143802	Low	Low	Low	Low	Low	Low

Appendix 3: Network maps and forest plots of outcomes

Figure S3.1: Network map of the effect on body weight, and forest plot of network effect sizes for comparison with placebo. The size of the nodes was proportional to the number of participants included in the trial, and the thickness of lines between the interventions relates to the number of studies.

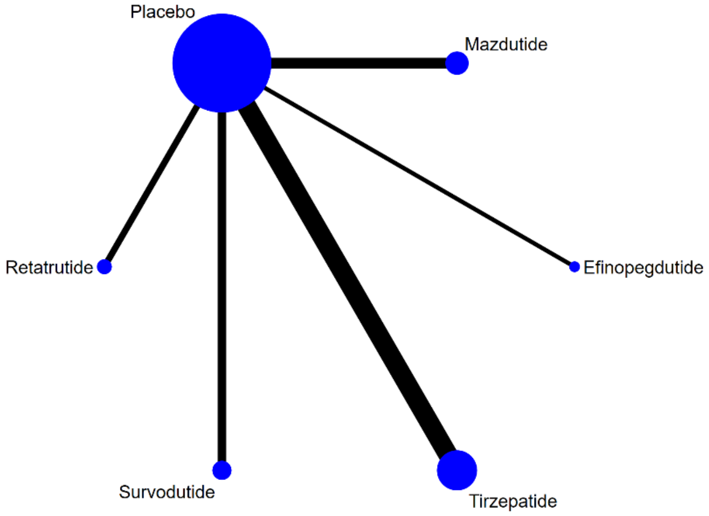

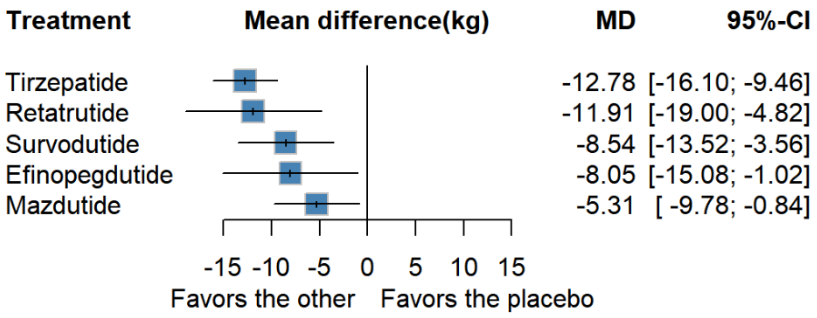

Figure S3.2: Network map of the effect on waist circumference., and forest plot of network effect sizes for comparison with placebo. The size of the nodes was proportional to the number of participants included in the trial, and the thickness of lines between the interventions relates to the number of studies.

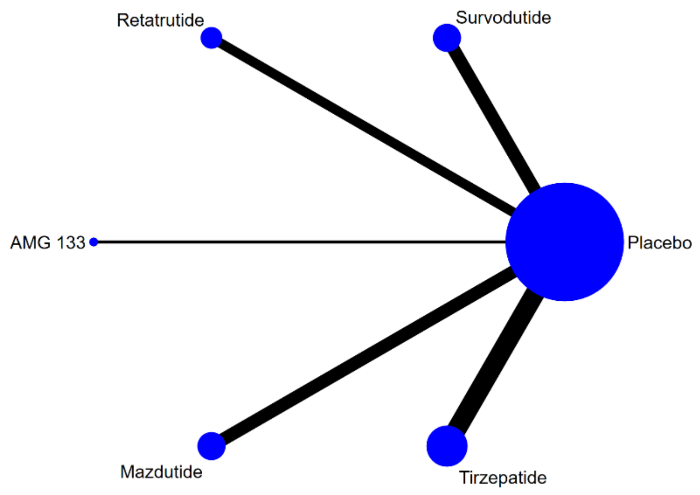

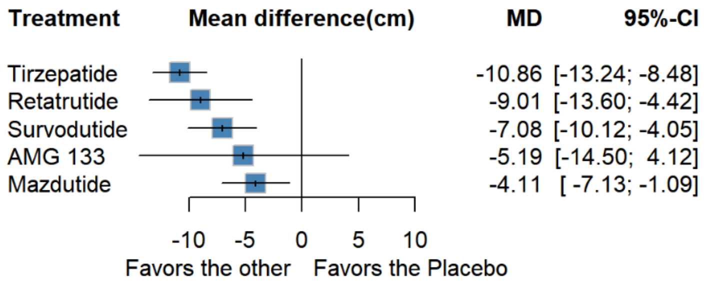

Figure S3.3: Network map of the effect on participants achieving a weight loss of more than 5%, and forest plot of network effect sizes for comparison with placebo. The size of the nodes was proportional to the number of participants included in the trial, and the thickness of lines between the interventions relates to the number of studies.

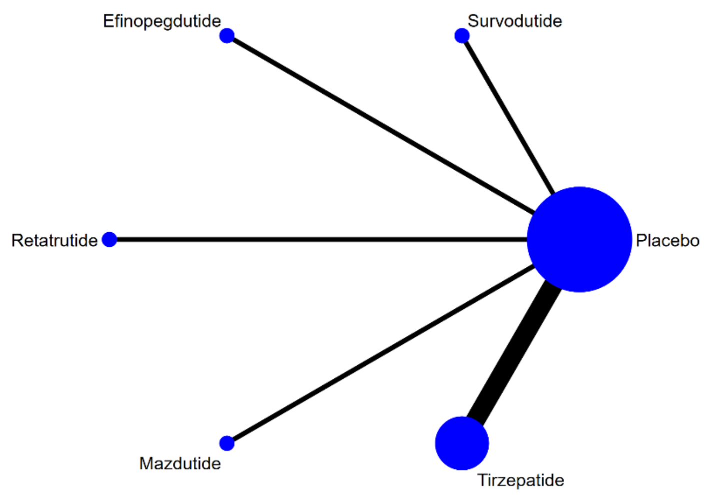

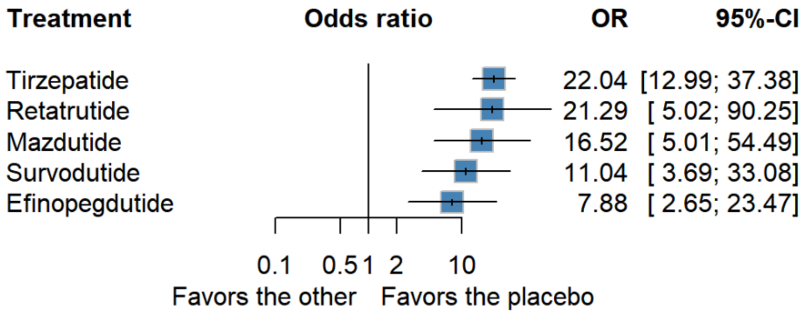

Figure S3.4: Network map of the effect on HbA_1c, and forest plot of network effect sizes for comparison with placebo. The size of the nodes was proportional to the number of participants included in the trial, and the thickness of lines between the interventions relates to the number of studies.

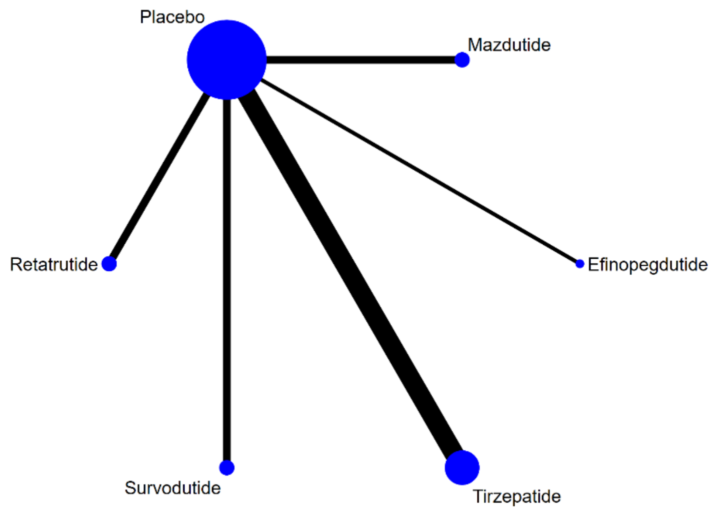

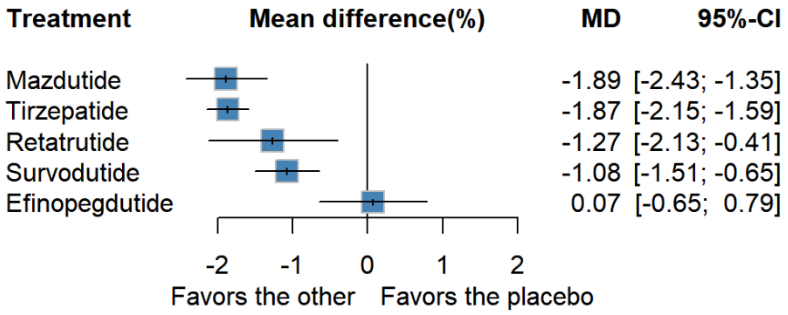

Figure S3.5: Network map of the effect on FPG, and forest plot of network effect sizes for comparison with placebo. The size of the nodes was proportional to the number of participants included in the trial, and the thickness of lines between the interventions relates to the number of studies.

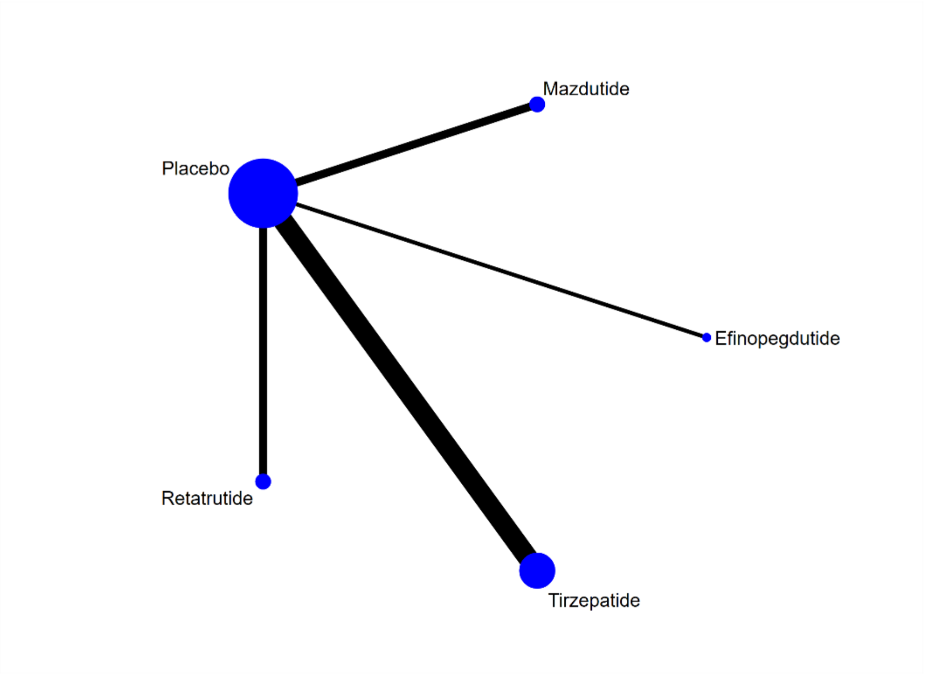

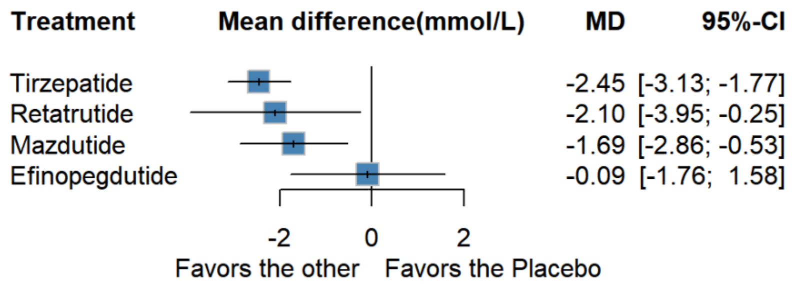

Figure S3.6: Network map of the effect on systolic blood pressure, and forest plot of network effect sizes for comparison with placebo. The size of the nodes was proportional to the number of participants included in the trial, and the thickness of lines between the interventions relates to the number of studies.

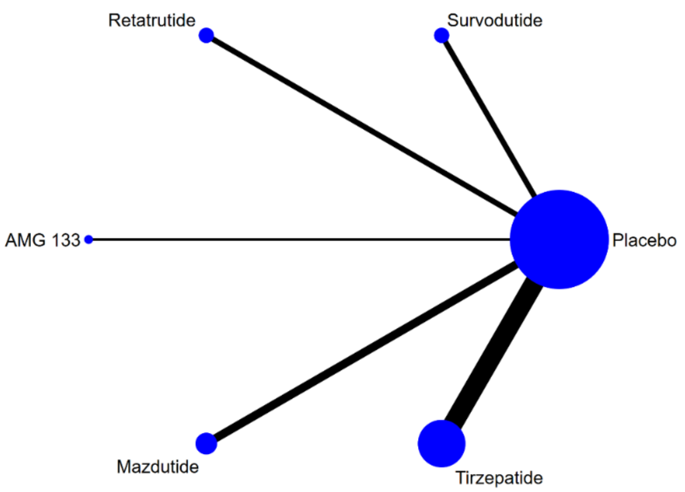

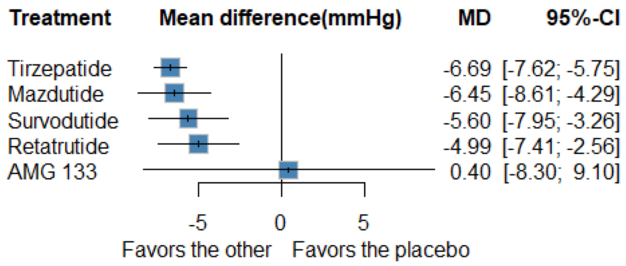

Figure S3.7: Network map of the effect on diastolic blood pressure, and forest plot of network effect sizes for comparison with placebo. The size of the nodes was proportional to the number of participants included in the trial, and the thickness of lines between the interventions relates to the number of studies.

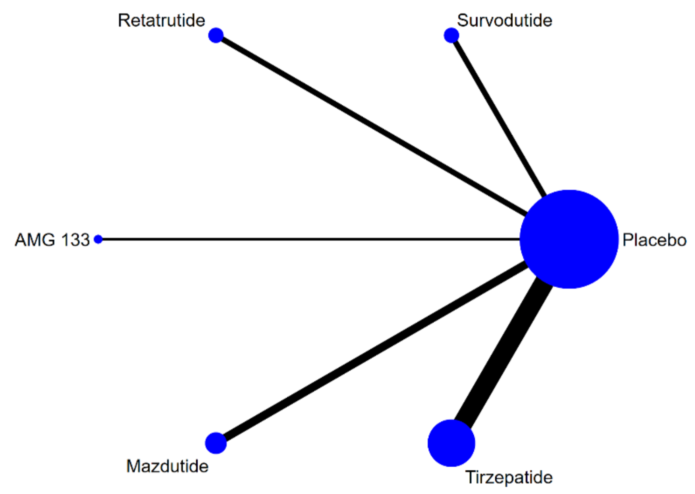

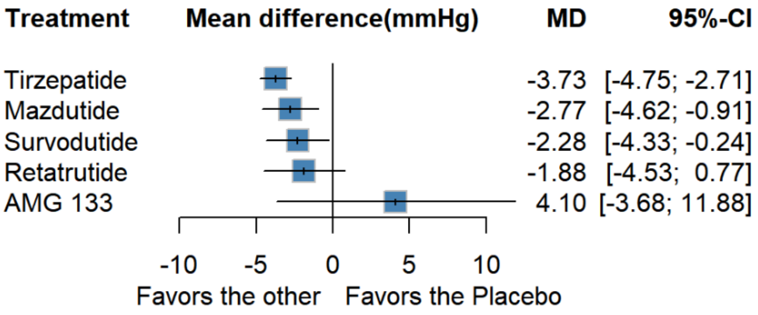

Figure S3.8: Network map of the effect on adverse effect, and forest plot of network effect sizes for comparison with placebo. The size of the nodes was proportional to the number of participants included in the trial, and the thickness of lines between the interventions relates to the number of studies.

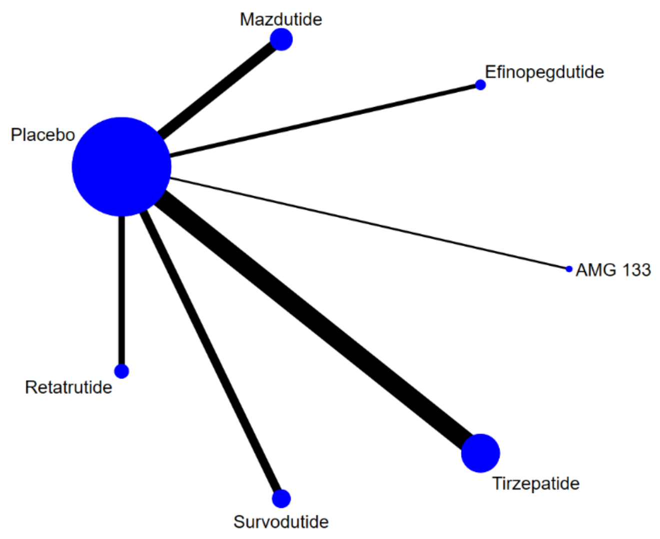

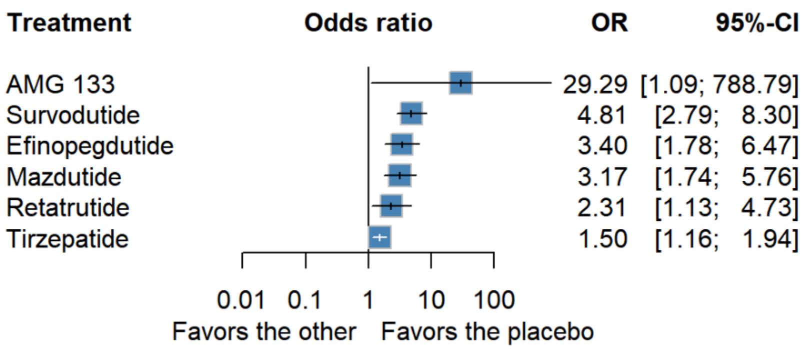

Figure S3.9: Network map of the effect on serious adverse effects, and forest plot of network effect sizes for comparison with placebo. The size of the nodes was proportional to the number of participants included in the trial, and the thickness of lines between the interventions relates to the number of studies.

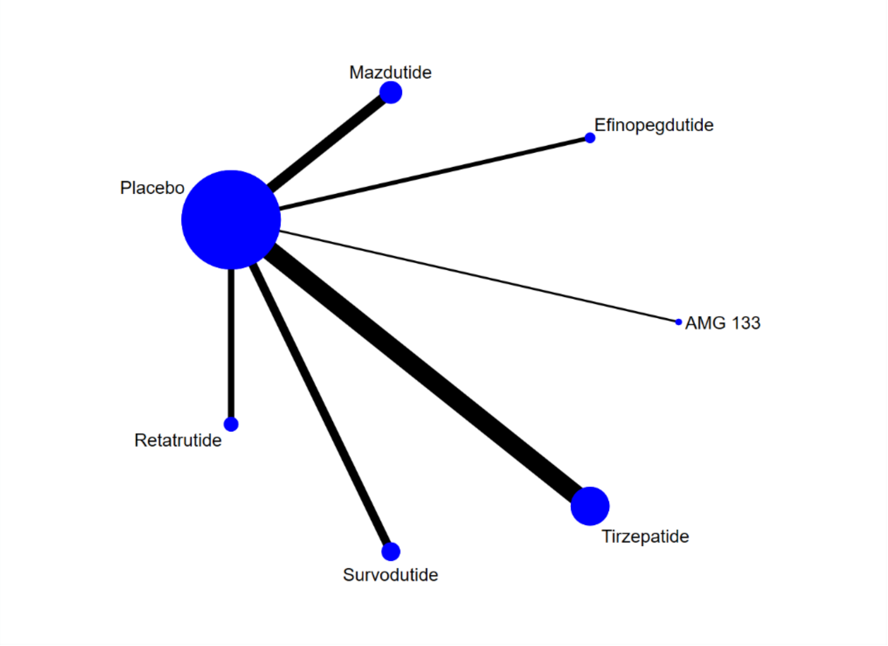

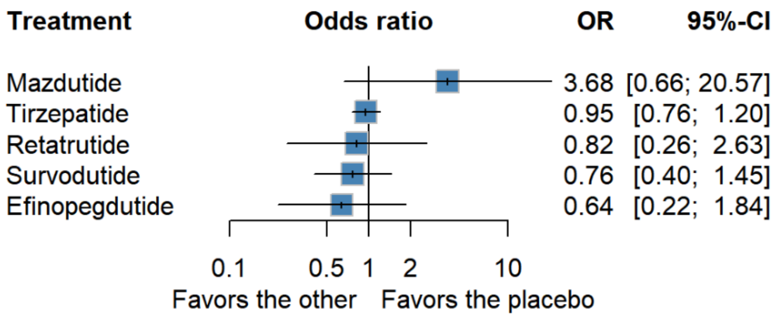

Figure S3.10: Network map of the effect on BMI, and forest plot of network effect sizes for comparison with placebo. The size of the nodes was proportional to the number of participants included in the trial, and the thickness of lines between the interventions relates to the number of studies.

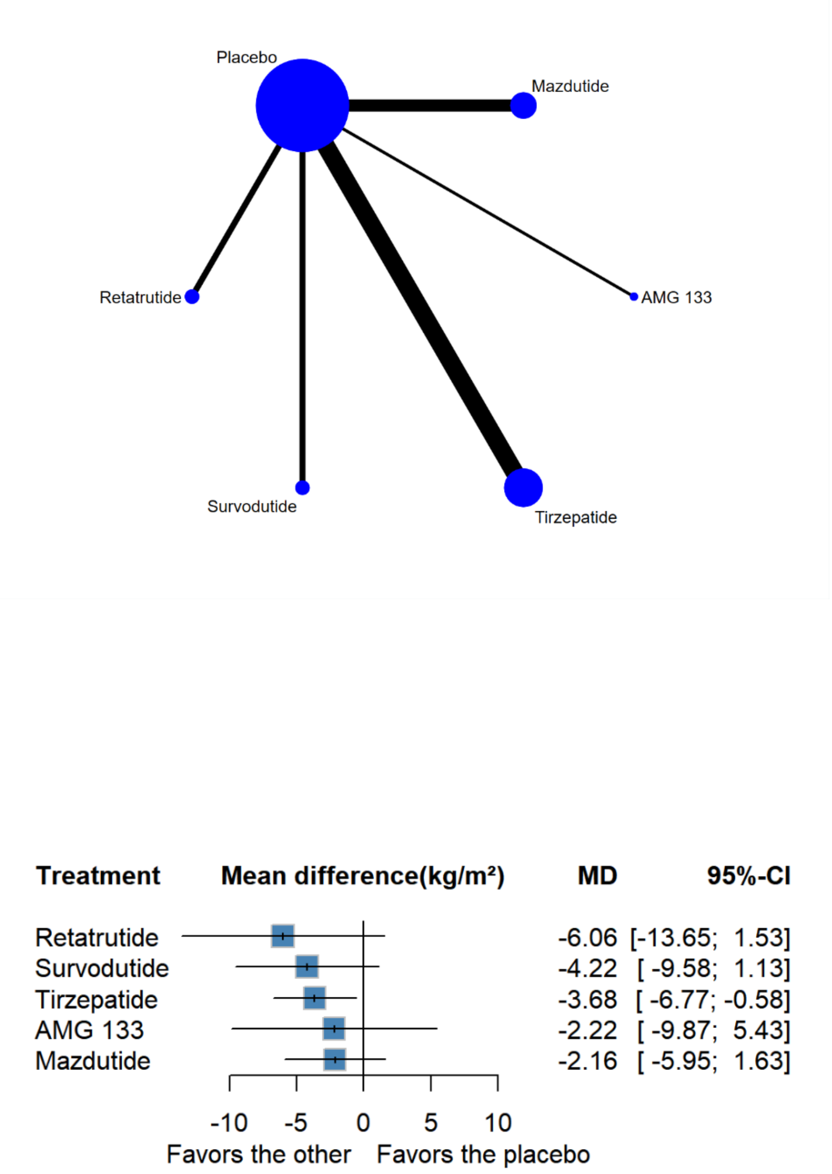

Figure S3.11: Network map of the effect on percent change of body weight, and forest plot of network effect sizes for comparison with placebo. The size of the nodes was proportional to the number of participants included in the trial, and the thickness of lines between the interventions relates to the number of studies.

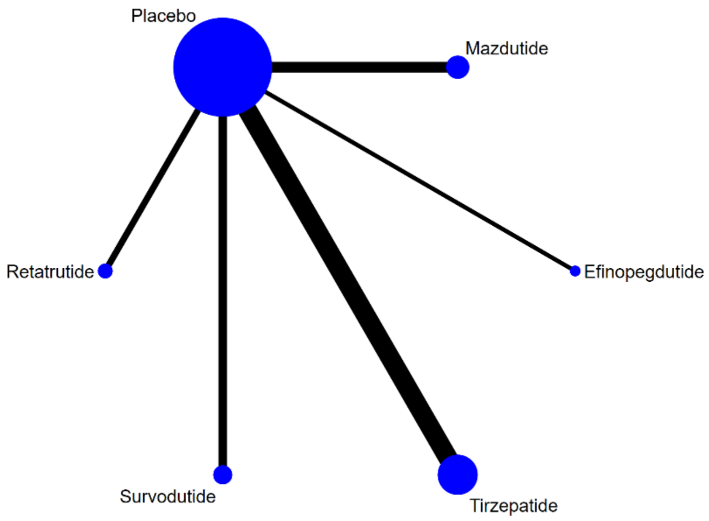

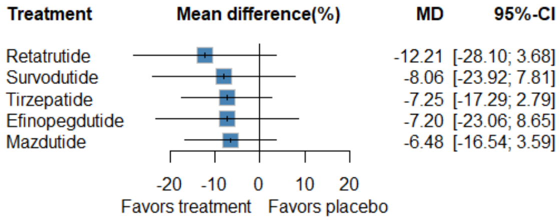

Appendix 4: Treatment ranking (P-scores) for the effects of various multi-receptor drugs

Table S4: Treatment ranking (P-scores) for the effects of various multi-receptor drugs

Body Weight		HbA$_1$c		FPG	
treatment	p-score	treatment	p-score	treatment	p-score
Tirzepatide	0.89	Mazdutide	0.88	Tirzepatide	0.87
Retatrutide	0.80	Tirzepatide	0.87	Retatrutide	0.73
Survodutide	0.59	Retatrutide	0.57	Mazdutide	0.61
Efinopegdutide	0.56	Survodutide	0.47	Efinopegdutide	0.17
Mazdutide	0.37	Placebo	0.12	Placebo	0.12
Placebo	0.08	Efinopegdutide	0.09		
**Waist Circumference**		**Weight Loss (≥ 5%)**		**Systolic Blood Pressure**	
treatment	p-score	treatment	p-score	treatment	p-score
Tirzepatide	0.92	Tirzepatide	0.80	Tirzepatide	0.84
Retatrutide	0.74	Retatrutide	0.74	Mazdutide	0.77
Survodutide	0.57	Mazdutide	0.65	Survodutide	0.60
AMG 133	0.43	Survodutide	0.47	Retatrutide	0.53
Mazdutide	0.31	Efinopegdutide	0.34	AMG 133	0.16
Placebo	0.03	Placebo	0.00	Placebo	0.11
**Diastolic Blood Pressure**		**Serious Adverse Effect**		**Adverse Effect**	
treatment	p-score	treatment	p-score	treatment	p-score
Tirzepatide	0.92	Efinopegdutide	0.75	Placebo	0.99
Mazdutide	0.70	Survodutide	0.68	Tirzepatide	0.80
Survodutide	0.60	Retatrutide	0.60	Retatrutide	0.59
Retatrutide	0.53	Tirzepatide	0.50	Mazdutide	0.43
Placebo	0.19	Placebo	0.41	Efinopegdutide	0.39
AMG 133	0.07	Mazdutide	0.06	Survodutide	0.21
				AMG 133	0.08
**BMI**					
treatment	p-score				
Retatrutide	0.78				
Survodutide	0.65				
Tirzepatide	0.61				
AMG133	0.43				
Mazdutide	0.42				
Placebo	0.11				

P-scores range from 0 to 1 to and can be interpreted as an average degree of probability for a treatment to be better than the other treatments in the network.

Appendix 5: League Table of Summary Estimates for Multi-receptor Drugs Derived from Network Meta-analysis of 24 Trials

Table S5.1: Body Weight

The columns represent the comparison of the row drug class to the column drug class. The rows represent the comparison of the row drug class to the column drug class. The effect estimates are expressed as mean difference and 95% confidence interval. Mean difference <0 favors the drug in the column, Mean difference >0 favors the drug in the row.

**Tirzepatide**					
-0.87<br>(-8.69 to 6.96)	**Retatrutide**				
-4.24<br>(-10.23 to 1.74)	-3.37<br>(-12.04 to 5.29)	**Survodutide**			
-4.73<br>(-12.50 to 3.05)	-3.86<br>(-13.85 to 6.12)	-0.49<br>(-9.10 to 8.13)	**Efinopegdutide**		
-7.47<br>(-13.04 to -1.90)	-6.60<br>(-14.98 to 1.77)	-3.23<br>(-9.92 to 3.46)	-2.74<br>(-11.07 to 5.59)	**Mazdutide**	
-12.78<br>(-16.10 to -9.46)	-11.91<br>(-19.00 to -4.82)	-8.54<br>(-13.52 to -3.56)	-8.05<br>(-15.08 to -1.02)	-5.31<br>(-9.78 to -0.84)	**Placebo**

Table S5.2: HbA$_1$c

The columns represent the comparison of the row drug class to the column drug class. The rows represent the comparison of the row drug class to the column drug class. The effect estimates are expressed as mean difference and 95% confidence interval. Mean difference <0 favors the drug in the column, Mean difference >0 favors the drug in the row.

**Mazdutide**					
-0.02<br>(-0.63 to 0.59)	**Tirzepatide**				
-0.62<br>(-1.64 to 0.40)	-0.60<br>(-1.51 to 0.31)	**Retatrutide**			
-0.81<br>(-1.51 to -0.12)	-0.79<br>(-1.30 to -0.28)	-0.19<br>(-1.16 to 0.77)	**Survodutide**		
-1.89<br>(-2.43 to -1.35)	-1.87<br>(-2.15 to -1.59)	-1.27<br>(-2.13 to -0.41)	-1.08<br>(-1.51 to -0.65)	**Placebo**	
-1.96<br>(-2.87 to -1.06)	-1.94<br>(-2.71 to -1.16)	-1.34<br>(-2.47 to -0.21)	-1.15<br>(-1.99 to -0.31)	-0.07<br>(-0.79 to 0.65)	**Efinopegdutide**

Table S5.3: FPG

The columns represent the comparison of the row drug class to the column drug class. The rows represent the comparison of the row drug class to the column drug class. The effect estimates are expressed as mean difference and 95% confidence interval. Mean difference <0 favors the drug in the column, Mean difference >0 favors the drug in the row.

Tirzepatide				
-0.35 (-2.32 to 1.62)	Retatrutide			
-0.76 (-2.11 to 0.59)	-0.41 (-2.59 to 1.78)	Mazdutide		
-2.36 (-4.16 to -0.56)	-2.01 (-4.50 to 0.48)	-1.60 (-3.64 to 0.43)	Efinopegdutide	
-2.45 (-3.13 to -1.77)	-2.10 (-3.95 to -0.25)	-1.69 (-2.86 to -0.53)	-0.09 (-1.76 to 1.58)	Placebo

Table S5.4: Waist Circumference

The columns represent the comparison of the row drug class to the column drug class. The rows represent the comparison of the row drug class to the column drug class. The effect estimates are expressed as mean difference and 95% confidence interval. Mean difference <0 favors the drug in the column, Mean difference >0 favors the drug in the row.

**Tirzepatide**					
-1.85 (-7.02 to 3.32)	**Retatrutide**				
-3.77 (-7.63 to 0.08)	-1.93 (-7.43 to 3.57)	**Survodutide**			
-5.67 (-15.28 to 3.95)	-3.82 (-14.20 to 6.56)	-1.89 (-11.69 to 7.90)	**AMG 133**		
-6.75 (-10.59 to -2.90)	-4.90 (-10.39 to 0.60)	-2.97 (-7.26 to 1.31)	-1.08 (-10.87 to 8.71)	**Mazdutide**	
-10.86 (-13.24 to -8.48)	-9.01 (-13.60 to -4.42)	-7.08 (-10.12 to -4.05)	-5.19 (-14.50 to 4.12)	-4.11 (-7.13 to -1.09)	**Placebo**

Table S5.5: Weight Loss (≥5%)

The columns represent the comparison of the row drug class to the column drug class. The rows represent the comparison of the row drug class to the column drug class. The effect estimates are expressed as odds ratio and 95% confidence interval. Odds ratio <1 favors the drug in the row, Odds ratio >1 favors the drug in the column.

**Tirzepatide**					
1.03 (0.22 to 4.76)	**Retatrutide**				
1.33 (0.36 to 5.00)	1.28 (0.20 to 8.33)	**Mazdutide**			
2.00 (0.59 to 6.67)	1.93 (0.31 to 11.83)	1.50 (0.30 to 7.57)	**Survodutide**		
2.78 (0.83 to 9.09)	2.70 (0.44 to 16.67)	2.08 (0.42 to 11.11)	1.41 (0.30 to 6.67)	**Efinopegdutide**	
22.04 (12.99 to 37.38)	21.29 (5.02 to 90.25)	16.52 (5.01 to 54.49)	11.04 (3.69 to 33.08)	7.88 (2.65 to 23.47)	**Placebo**

Table S5.6: Systolic Blood Pressure

The columns represent the comparison of the row drug class to the column drug class. The rows represent the comparison of the row drug class to the column drug class. The effect estimates are expressed as mean difference and 95% confidence interval. Mean difference <0 favors the drug in the column, Mean difference >0 favors the drug in the row.

Tirzepatide					
-0.24<br>(-2.59 to 2.12)	Mazdutide				
-1.08<br>(-3.61 to 1.44)	-0.85<br>(-4.04 to 2.34)	Survodutide			
-1.70<br>(- 4.30 to 0.90)	-1.47<br>(-4.71 to 1.78)	-0.62<br>(-3.99 to 2.76)	Retatrutide		
-6.69<br>(-7.62 to -5.75)	-6.45<br>(-8.61 to -4.29)	-5.60<br>(-7.95 to -3.26)	-4.99<br>(-7.41 to -2.56)	Placebo	
-7.09<br>(-15.84 to 1.67)	-6.85<br>(-15.82 to -2.12)	-6.00<br>(- 15.02 to 3.01)	-5.39<br>(-14.42 to 3.65)	-0.40<br>(-9.10 to 8.30)	AMG 133

Table S5.7: Diastolic Blood Pressure

The columns represent the comparison of the row drug class to the column drug class. The rows represent the comparison of the row drug class to the column drug class. The effect estimates are expressed as mean difference and 95% confidence interval. Mean difference <0 favors the drug in the column, Mean difference >0 favors the drug in the row.

**Tirzepatide**					
-0.96<br>(-3.07 to 1.15)	**Mazdutide**				
-1.44<br>(-3.73 to 0.85)	-0.48<br>(-3.24 to 2.28)	**Survodutide**			
-1.85<br>(-4.68 to 0.99)	-0.89<br>(-4.11 to 2.34)	-0.40<br>(-3.75 to 2.94)	**Retatrutide**		
-3.73<br>(-4.75 to -2.71)	-2.77<br>(-4.62 to -0.91)	-2.28<br>(-4.33 to -0.24)	-1.88<br>(-4.53 to 0.77)	**Placebo**	
-7.83<br>(-15.68 to 0.02)	-6.87<br>(-14.87 to 1.13)	-6.38<br>(-14.43 to 1.66)	-5.98<br>(-14.20 to 2.24)	-4.10<br>(-11.88 to 3.68)	**AMG 133**

Table S5.8: Serious Adverse Effect

The columns represent the comparison of the row drug class to the column drug class. The rows represent the comparison of the row drug class to the column drug class. The effect estimates are expressed as odds ratio and 95% confidence interval. Odds ratio <1 favors the drug in the column, Odds ratio >1 favors the drug in the row.

**Mazdutide**					
3.86<br>(0.68 to 21.92)	**Tirzepatide**				
4.48<br>(0.56 to 35.77)	1.16<br>(0.35 to 3.85)	**Retatrutide**			
4.83<br>(0.77 to 30.24)	1.25<br>(0.63 to 2.5)	1.08<br>(0.29 to 4.06)	**Survodutide**		
5.88<br>(0.76 to 50)	1.49<br>(0.51 to 4.35)	1.28<br>(0.27 to 6.25)	1.19<br>(0.35 to 4.17)	**Efinopegdutide**	
3.68<br>(0.66 to 20.57)	0.95<br>(0.76 to 1.20)	0.82<br>(0.26 to 2.63)	0.76<br>(0.40 to 1.45)	0.64<br>(0.22 to 1.84)	**Placebo**

Table S5.8: Adverse Effect

The columns represent the comparison of the row drug class to the column drug class. The rows represent the comparison of the row drug class to the column drug class. The effect estimates are expressed as odds ratio and 95% confidence interval. Odds ratio <1 favors the drug in the column, Odds ratio >1 favors the drug in the row.

**AMG 133**						
6.08<br>(0.22 to 171.34)	**Survodutide**					
8.62<br>(0.30 to 247.03)	1.41<br>(0.62 to 3.33)	**Efinopegdutide**				
9.24<br>(0.33 to 262.59)	1.52<br>(0.68 to 3.45)	1.07<br>(0.45 to 2.58)	**Mazdutide**			
12.66<br>(0.44 to 368.15)	2.08<br>(0.85 to 5.00)	1.47<br>(0.56 to 3.85)	1.37<br>(0.54 to 3.48)	**Retatrutide**		
19.50<br>(0.72 to 530.41)	3.21<br>(1.76 to 5.85)	2.26<br>(1.13 to 4.53)	2.11<br>(1.10 to 4.04)	1.54<br>(0.72 to 3.29)	**Tirzepatide**	
29.29<br>(1.09 to 788.79)	4.81<br>(2.79 to 8.30)	3.40<br>(1.78 to 6.47)	3.17<br>(1.74 to 5.76)	2.31<br>(1.13 to 4.73)	1.5<br>(1.16 to 1.94)	**Placebo**

Table S5.7: BMI

The columns represent the comparison of the row drug class to the column drug class. The rows represent the comparison of the row drug class to the column drug class. The effect estimates are expressed as mean difference and 95% confidence interval. Mean difference <0 favors the drug in the column, Mean difference >0 favors the drug in the row.

Retatrutide					
-1.84<br>(-11.12 to 7.45)	Survodutide				
-2.38<br>(-10.58 to 5.81)	-0.55<br>(-6.73 to 5.64)	Tirzepatide			
-3.84<br>(-14.61 to 6.93)	-2.00<br>(-11.34 to 7.33)	-1.46<br>(-9.71 to 6.80)	AMG 133		
-3.90<br>(-12.38 to 4.58)	-2.06<br>(-8.62 to 4.50)	-1.51<br>(-6.40 to 3.38)	-0.06<br>(-8.60 to 8.48)	Mazdutide	
-6.06<br>(-13.65 to 1.53)	-4.22<br>(-9.58 to 1.13)	-3.68<br>(-6.77 to -0.58)	-2.22<br>(-9.87 to 5.43)	-2.16<br>(-5.95 to 1.63)	Placebo

Appendix 6: CINeMA Assessment

Table S6.1: CINeMA Results of HbA_1c

Comparison	Within-study bias	Reporting bias	Indirectness	Imprecision	Heterogeneity	Incoherence	Confidence rating
Placebo:Survodutide	No concerns	Low risk	No concerns	No concerns	No concerns	No concerns	High
Placebo:Efinopegdutide	Some concerns	Low risk	Some concerns	Major concerns	No concerns	No concerns	Low
Placebo:Retatrutide	No concerns	Low risk	No concerns	No concerns	No concerns	No concerns	High
Placebo:Mazdutide	No concerns	Low risk	No concerns	No concerns	No concerns	No concerns	High
Placebo:Tirzepatide	No concerns	Low risk	No concerns	No concerns	No concerns	No concerns	High
Survodutide:Efinopegdutide	Some concerns	Low risk	No concerns	No concerns	Major concerns	No concerns	Low
Survodutide:Retatrutide	No concerns	Low risk	No concerns	Major concerns	No concerns	No concerns	Low
Survodutide:Mazdutide	No concerns	Low risk	No concerns	No concerns	Major concerns	No concerns	Low
Survodutide:Tirzepatide	No concerns	Low risk	No concerns	No concerns	Major concerns	No concerns	Low
Efinopegdutide:Retatrutide	No concerns	Low risk	No concerns	No concerns	No concerns	No concerns	High

Efinopegdutide:Mazdutide	Some concerns	Low risk	No concerns	No concerns	No concerns	No concerns	Moderate
Efinopegdutide:Tirzepatide	Some concerns	Low risk	Some concerns	No concerns	No concerns	No concerns	Moderate
Retatrutide:Mazdutide	No concerns	Low risk	No concerns	Major concerns	No concerns	No concerns	Low
Retatrutide:Tirzepatide	No concerns	Low risk	No concerns	Major concerns	No concerns	No concerns	Low
Mazdutide:Tirzepatide	No concerns	Low risk	No concerns	Major concerns	No concerns	No concerns	Low

Table S6.2: CINeMA Results of FPG

Comparison	Within-study bias	Reporting bias	Indirectness	Imprecision	Heterogeneity	Incoherence	Confidence rating
Placebo:Efinopegdutide	No concerns	Low risk	No concerns	No concerns	Major concerns	No concerns	Low
Placebo:Retatrutide	No concerns	Low risk	No concerns	No concerns	Some concerns	No concerns	Moderate
Placebo:Mazdutide	No concerns	Low risk	No concerns	No concerns	Some concerns	No concerns	Moderate
Placebo:Tirzepatide	No concerns	Low risk	No concerns	No concerns	No concerns	No concerns	High
Efinopegdutide:Retatrutide	No concerns	Low risk	No concerns	Some concerns	No concerns	No concerns	Moderate
Efinopegdutide:Mazdutide	No concerns	Low risk	No concerns	Some concerns	No concerns	No concerns	Moderate
Efinopegdutide:Tirzepatide	No concerns	Low risk	No concerns	No concerns	Some concerns	No concerns	Moderate
Retatrutide:Mazdutide	No concerns	Low risk	No concerns	No concerns	Major concerns	No concerns	Low
Retatrutide:Tirzepatide	No concerns	Low risk	No concerns	Some concerns	Some concerns	No concerns	Moderate
Mazdutide:Tirzepatide	No concerns	Low risk	No concerns	Some concerns	No concerns	No concerns	Moderate

Table S6.3: CINeMA Results of body weight (With T2D)

Comparison	Within-study bias	Reporting bias	Indirectness	Imprecision	Heterogeneity	Incoherence	Confidence rating
Placebo:Survodutide	Some concerns	Low risk	Some concerns	No concerns	Some concerns	No concerns	Moderate
Placebo:Efinopegdutide	No concerns	Low risk	No concerns	No concerns	Some concerns	No concerns	Moderate
Placebo:Retatrutide	No concerns	Low risk	Some concerns	No concerns	Some concerns	No concerns	Moderate
Placebo:Mazdutide	No concerns	Low risk	No concerns	Some concerns	Some concerns	No concerns	Moderate
Placebo:Tirzepatide	No concerns	Low risk	No concerns	No concerns	No concerns	No concerns	High
Survodutide:Efinopegdutide	No concerns	Low risk	No concerns	Major concerns	No concerns	No concerns	Low
Survodutide:Retatrutide	No concerns	Low risk	Some concerns	Major concerns	No concerns	No concerns	Low
Survodutide:Mazdutide	No concerns	Low risk	No concerns	No concerns	Some concerns	No concerns	Moderate
Survodutide:Tirzepatide	No concerns	Low risk	No concerns	Some concerns	Some concerns	No concerns	Moderate
Efinopegdutide:Retatrutide	No concerns	Low risk	No concerns	Major concerns	No concerns	No concerns	Low
Efinopegdutide:Mazdutide	No concerns	Low risk	No concerns	Some concerns	Some concerns	No concerns	Moderate

Efinopegdutide:Tirzepatide	No concerns	Low risk	No concerns	Some concerns	Some concerns	No concerns	Moderate
Retatrutide:Mazdutide	No concerns	Low risk	No concerns	Some concerns	Some concerns	No concerns	Moderate
Retatrutide:Tirzepatide	No concerns	Low risk	No concerns	Some concerns	Some concerns	No concerns	Moderate
Mazdutide:Tirzepatide	No concerns	Low risk	No concerns	No concerns	Some concerns	No concerns	Moderate

Table S6.4: CINeMA Results of body weight (Without T2D)

Comparison	Within-study bias	Reporting bias	Indirectness	Imprecision	Heterogeneity	Incoherence	Confidence rating
Placebo:Survodutide	No concerns	Low risk	No concerns	No concerns	Some concerns	No concerns	Moderate
Placebo:Efinopegdutide	Major concerns	Low risk	Some concerns	No concerns	Some concerns	No concerns	Low
Placebo:Retatrutide	Major concerns	Low risk	Some concerns	No concerns	No concerns	No concerns	Low
Placebo:Mazdutide	No concerns	Low risk	No concerns	No concerns	Some concerns	No concerns	Moderate
Placebo:Tirzepatide	No concerns	Low risk	No concerns	No concerns	No concerns	No concerns	High
Survodutide:Efinopegdutide	Some concerns	Low risk	No concerns	Major concerns	No concerns	No concerns	Low
Survodutide:Retatrutide	Some concerns	Low risk	No concerns	No concerns	Major concerns	No concerns	Low
Survodutide:Mazdutide	No concerns	Low risk	No concerns	Some concerns	Some concerns	No concerns	Moderate
Survodutide:Tirzepatide	No concerns	Low risk	No concerns	No concerns	Some concerns	No concerns	Moderate
Efinopegdutide:Retatrutide	Major concerns	Low risk	Some concerns	Some concerns	Some concerns	No concerns	Low
Efinopegdutide:Mazdutide	Some concerns	Low risk	Some concerns	Major concerns	No concerns	No concerns	Low

Efinopegdutide:Tirzepatide	Some concerns	Low risk	Some concerns	No concerns	Some concerns	No concerns	Moderate
Retatrutide:Mazdutide	Some concerns	Low risk	Some concerns	No concerns	Some concerns	No concerns	Moderate
Retatrutide:Tirzepatide	Some concerns	Low risk	Some concerns	Major concerns	No concerns	No concerns	Low
Mazdutide:Tirzepatide	No concerns	Low risk	No concerns	No concerns	No concerns	No concerns	High

Appendix 7: Funnel plots

The Figure show the assessment of small study effect bias in studies on the effects of various multi-receptor drugs comparing. The funnel plots pertain to all trials comparing GLP-1 receptor co-agonists versus placebo. We also used Egger's test to assess the symmetry of the funnel plot, if P > 0.05, the funnel plot is proved to be symmetrical and may not have publication bias.

Figure S7.1 Funnel plot for the change from baseline in HbA1c (%) for patients with T2D.

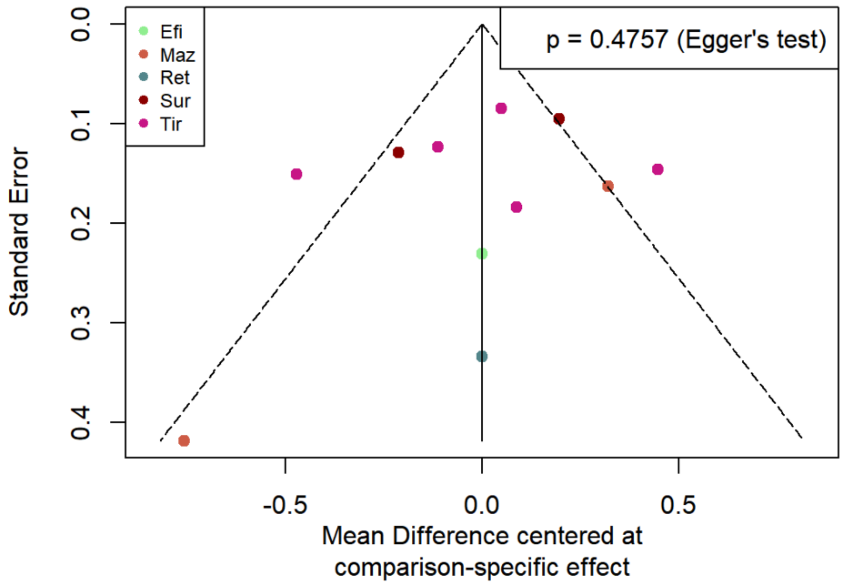

Figure S7.2 Funnel plot for the change from baseline in body weight (kg).

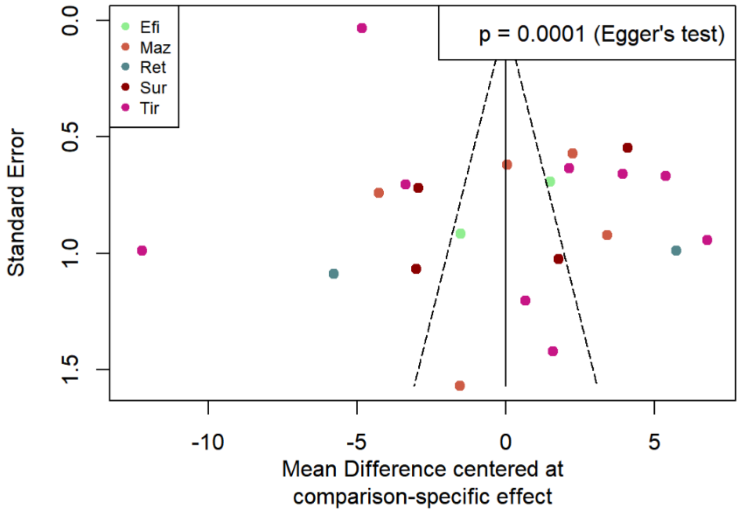

Figure S7.2a Funnel plot for the change from baseline in body weight (kg) for patients with T2D.

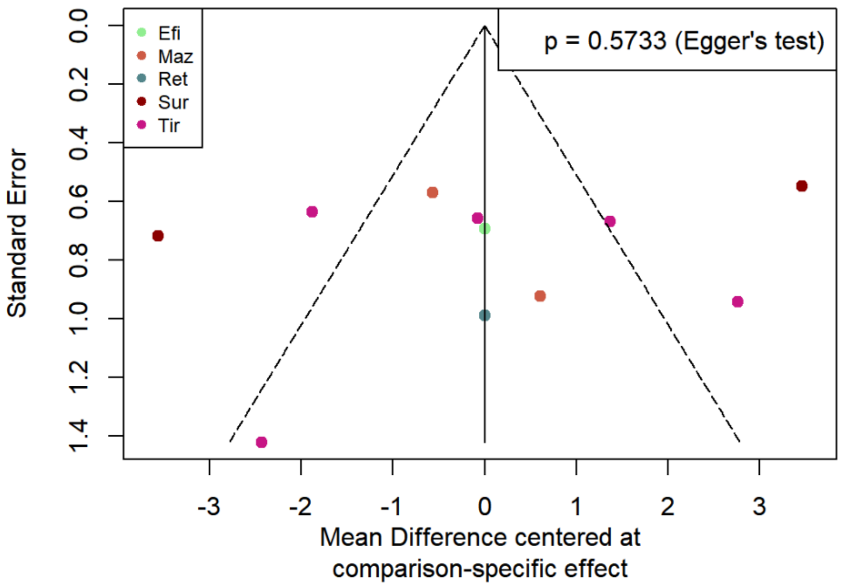

Figure S7.2b Funnel plot for the change from baseline in body weight (kg) for patients without T2D.

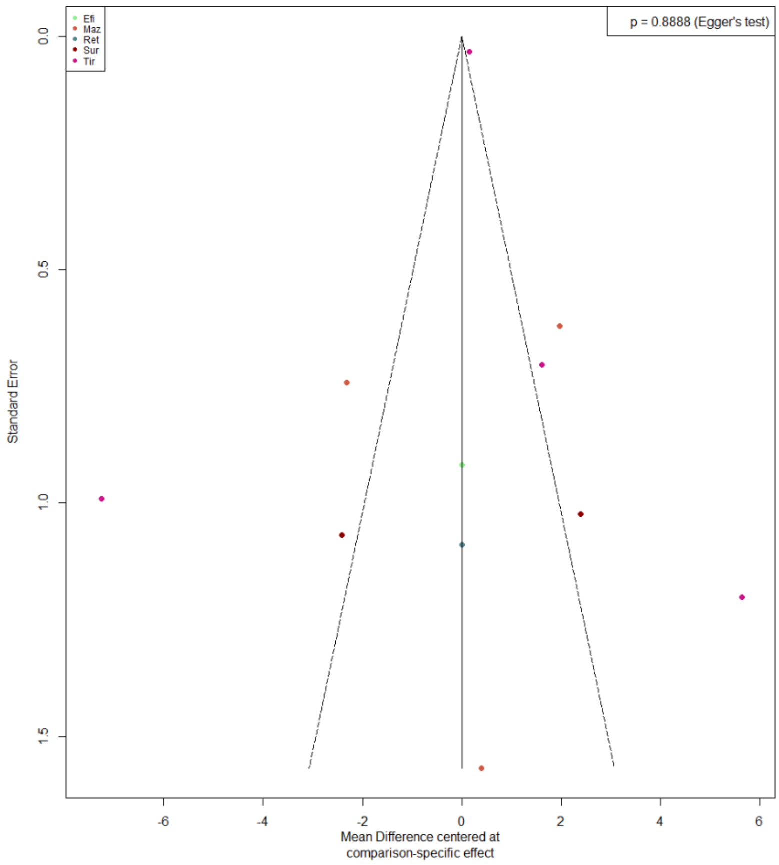

Figure S7.3 Funnel plot for the change from baseline in Waist Circumference (cm).

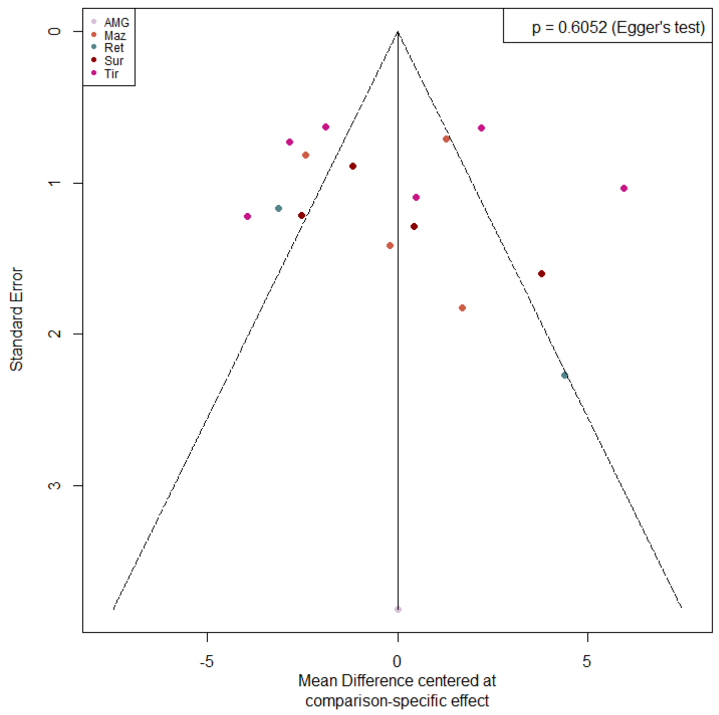

Figure S7.4 Funnel plot for the change from baseline in weight loss (≥5%)

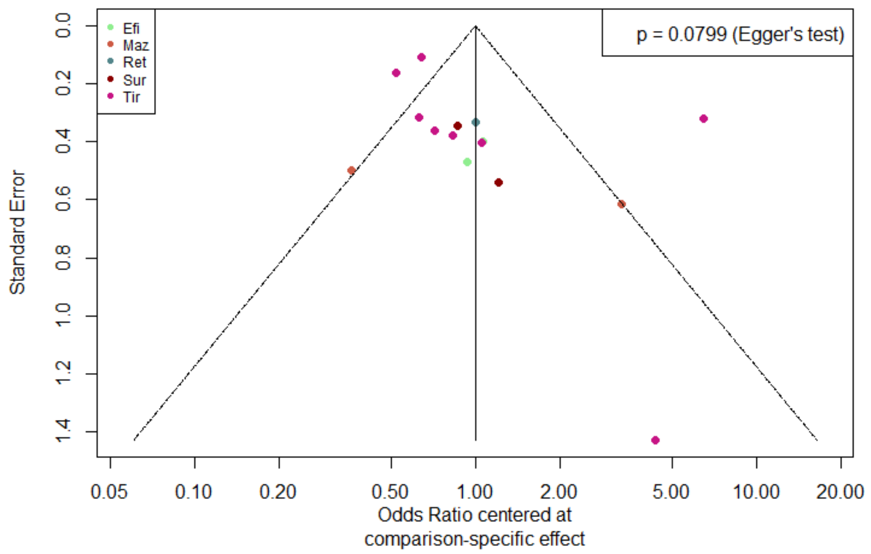

Figure S7.5 Funnel plot for the change from baseline in systolic blood pressure (mmHg)

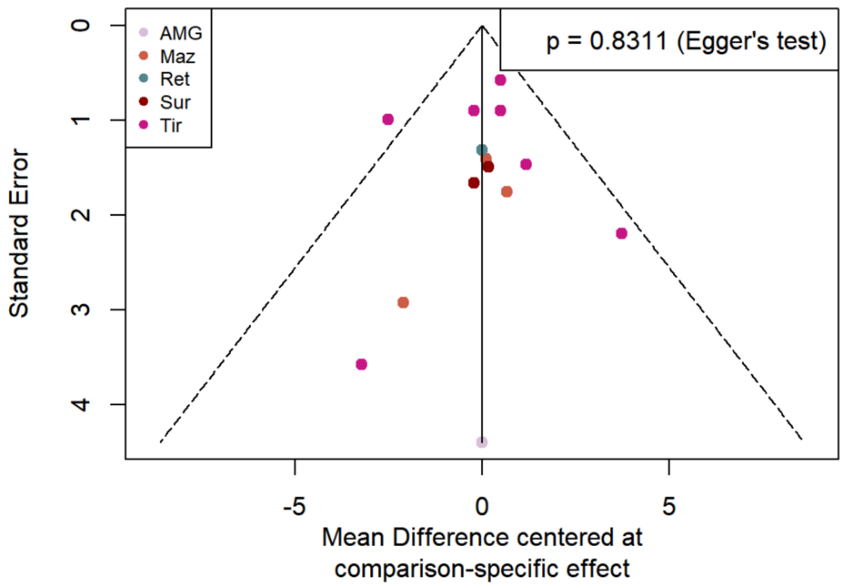

Figure S7.6 Funnel plot for the change from baseline in diastolic blood pressure (mmHg)

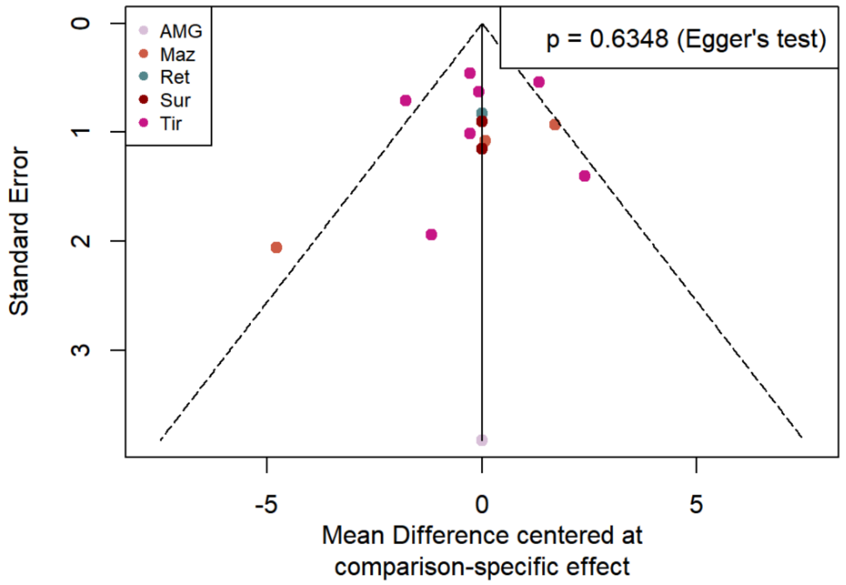

Figure S7.7 Funnel plot for the change from baseline in serious adverse effect

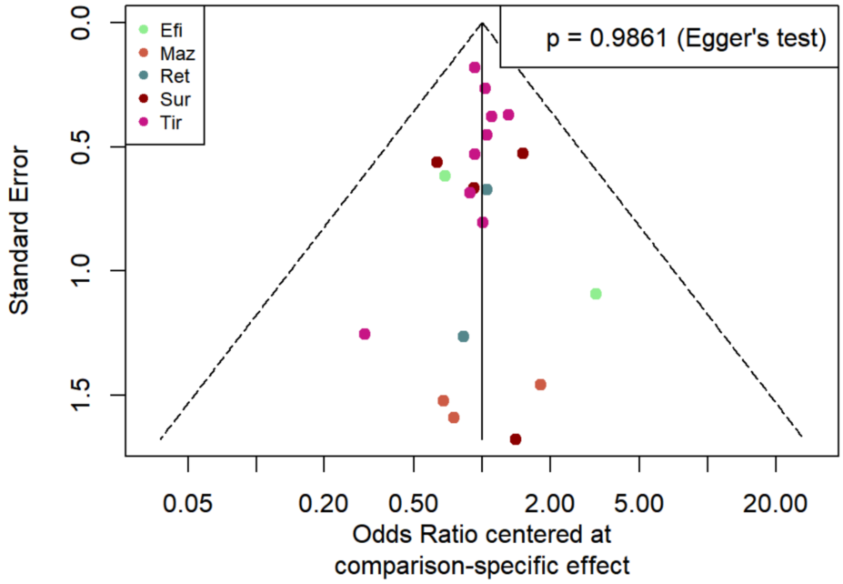

Figure S7.8 Funnel plot for the change from baseline in adverse effect.

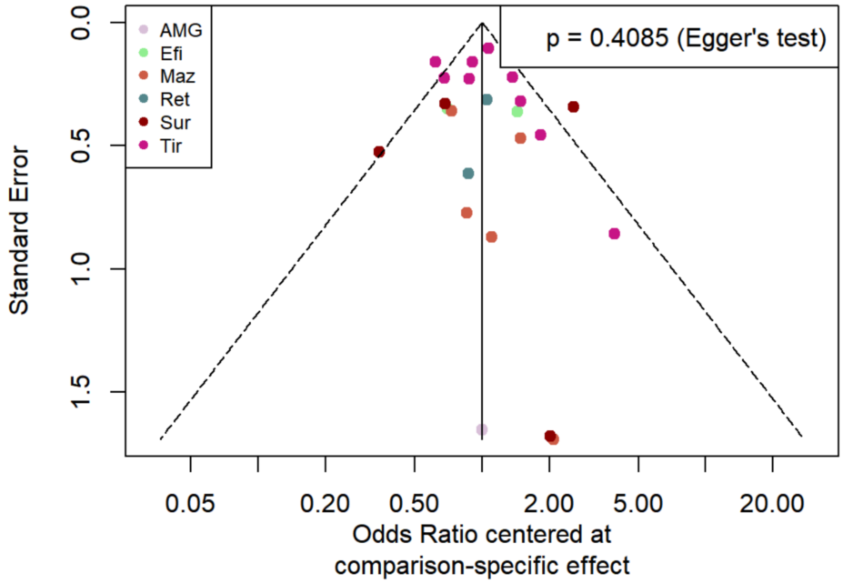

Figure S7. Funnel plot for the change from baseline in BMI.

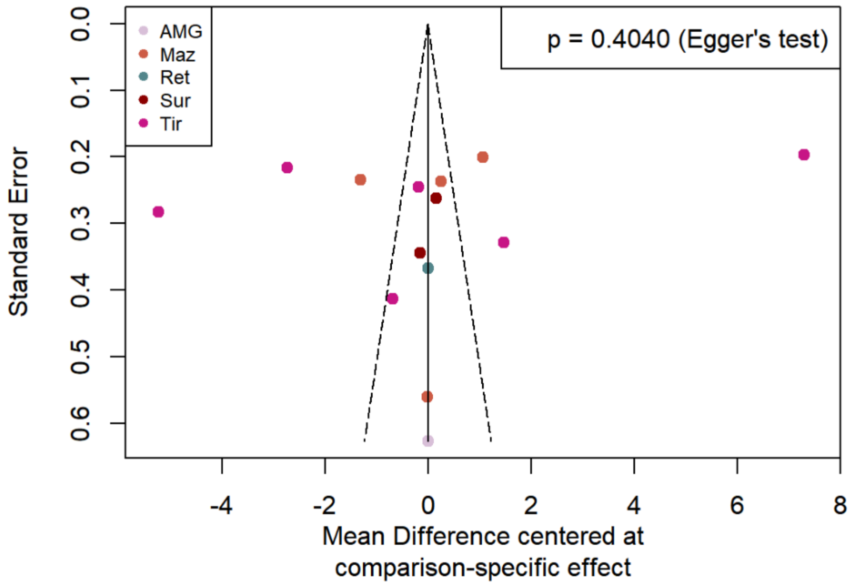

Figure S8. Funnel plot for the change from baseline in percent change of body weight.

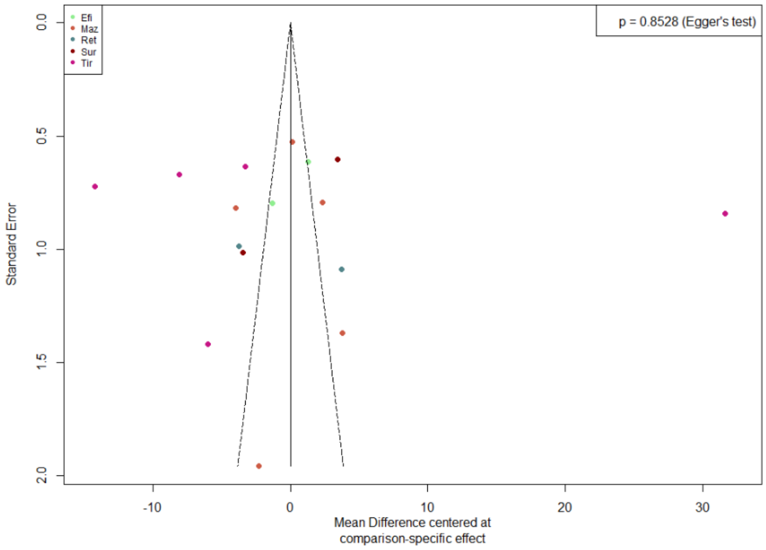

Appendix 8: Subgroup analysis of multi-receptor drugs on patients with or without T2D

Figure S8.1: Network map of the effect on body weight (patients with T2D), and forest plot of network effect sizes for comparison with placebo. The size of the nodes was proportional to the number of participants included in the trial, and the thickness of lines between the interventions relates to the number of studies.

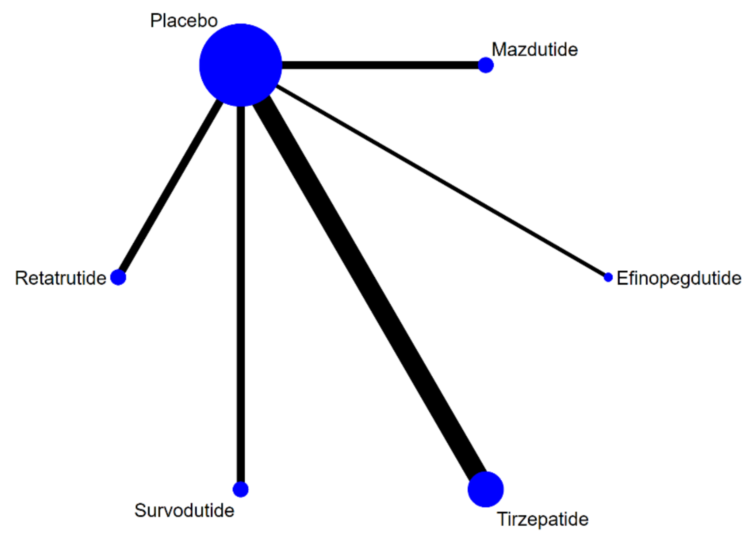

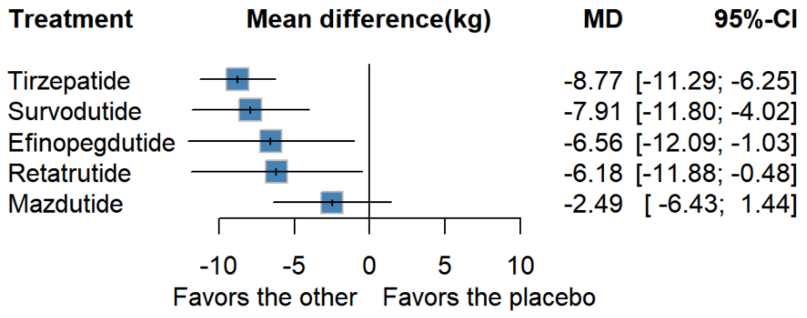

Figure S8.2: Network map of the effect on body weight (patients without T2D), and forest plot of network effect sizes for comparison with placebo. The size of the nodes was proportional to the number of participants included in the trial, and the thickness of lines between the interventions relates to the number of studies.

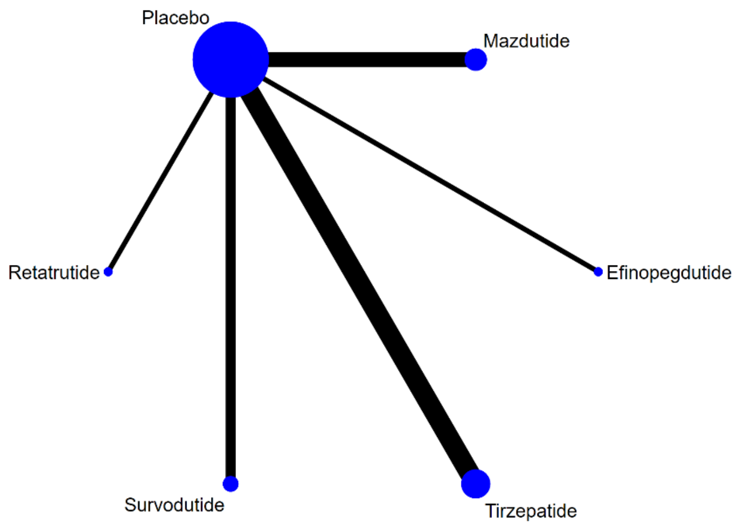

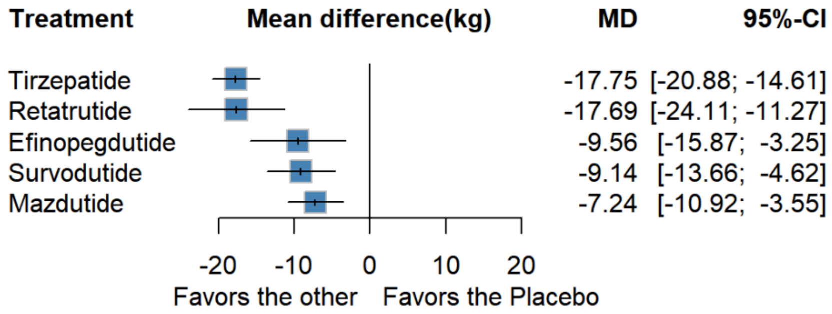

Figure S8.3: Network map of the effect on participants achieving a weight loss of more than 5% (patients with T2D), and forest plot of network effect sizes for comparison with placebo. The size of the nodes was proportional to the number of participants included in the trial, and the thickness of lines between the interventions relates to the number of studies.

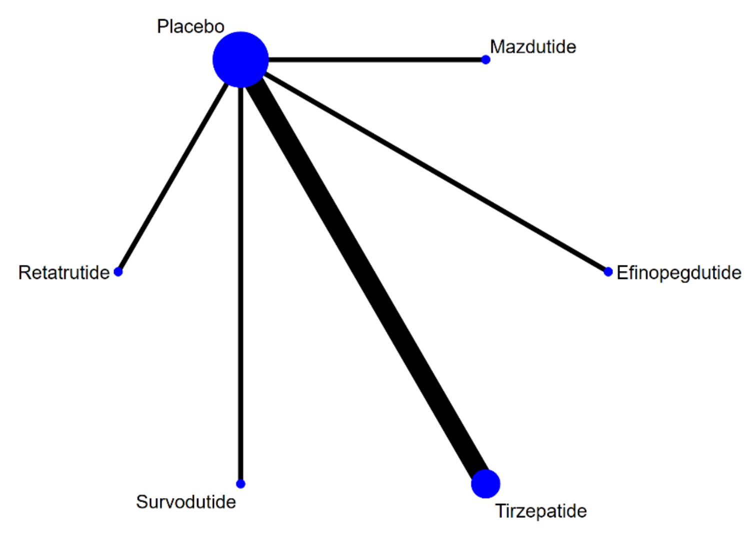

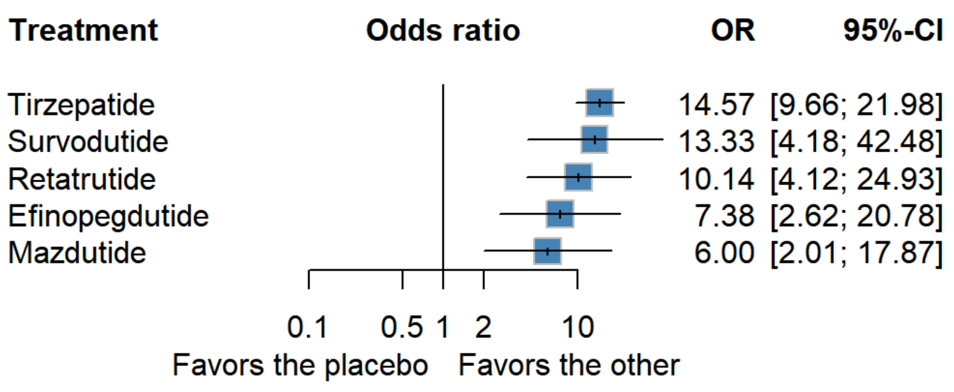

Figure S8.4: Network map of the effect on participants achieving a weight loss of more than 5% (patients without T2D), and forest plot of network effect sizes for comparison with placebo. The size of the nodes was proportional to the number of participants included in the trial, and the thickness of lines between the interventions relates to the number of studies.

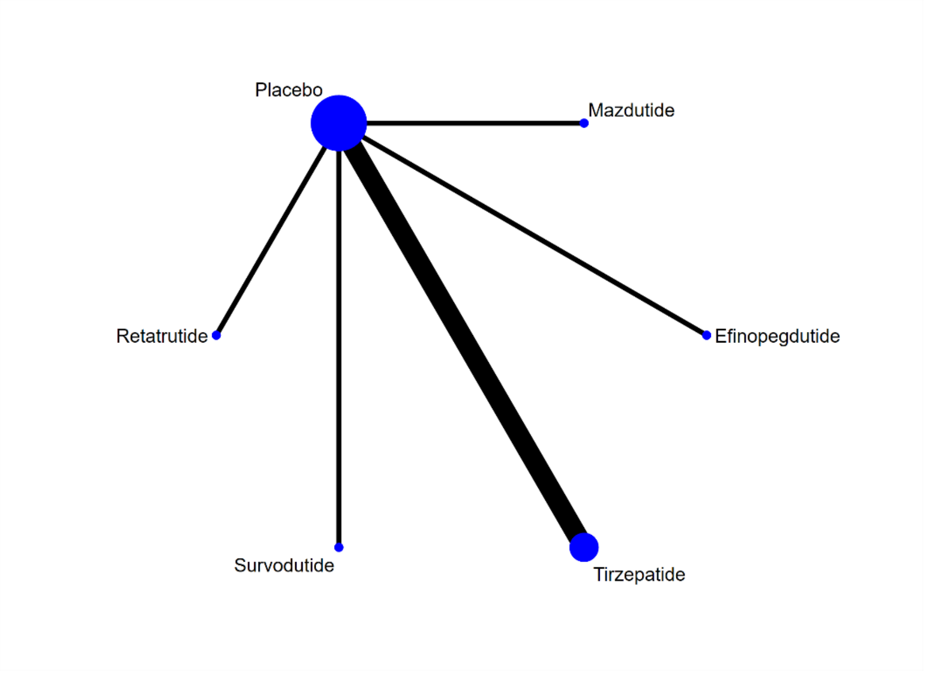

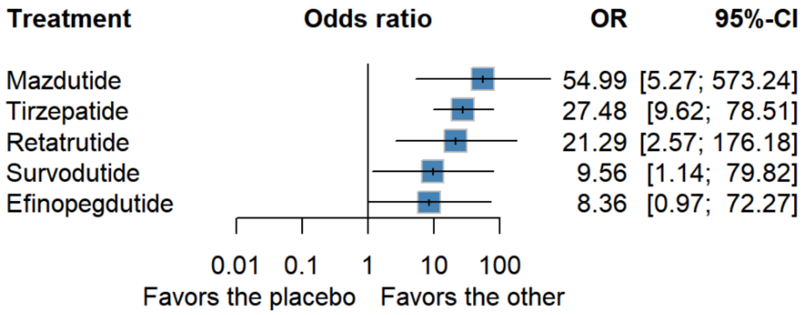

Figure S8.5: Network map of the effect on systolic blood pressure (patients with T2D), and forest plot of network effect sizes for comparison with placebo. The size of the nodes was proportional to the number of participants included in the trial, and the thickness of lines between the interventions relates to the number of studies.

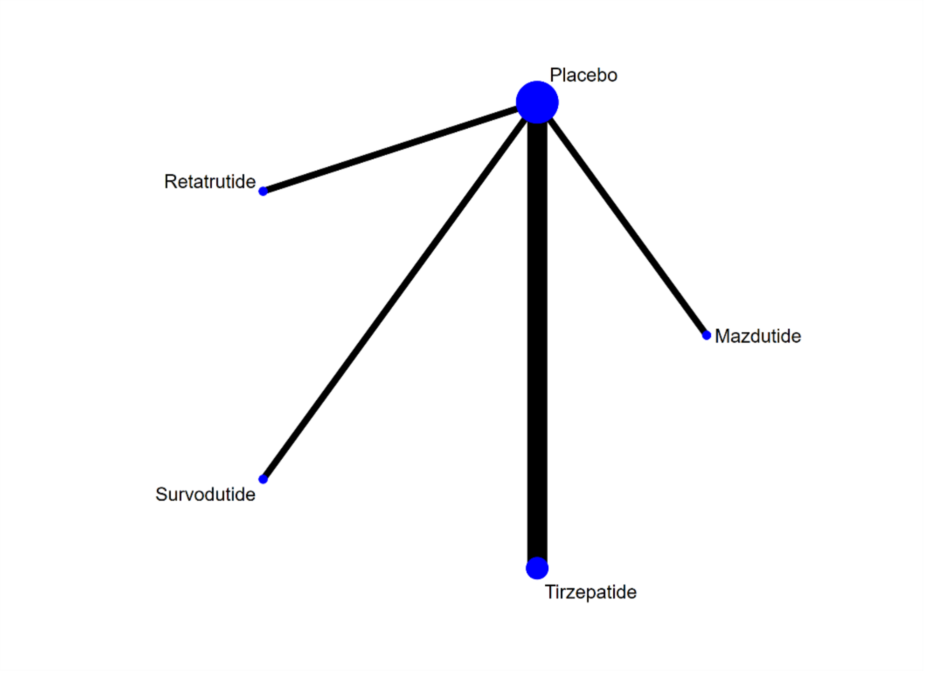

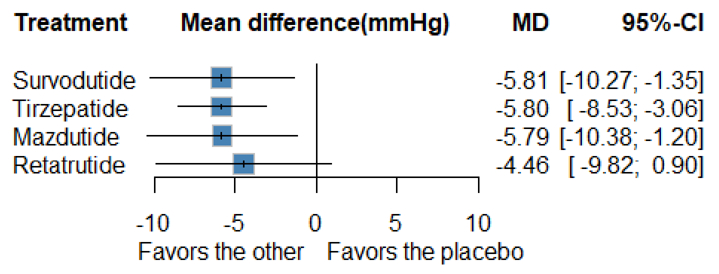

Figure S8.6: Network map of the effect on diastolic blood pressure (patients with T2D), and forest plot of network effect sizes for comparison with placebo. The size of the nodes was proportional to the number of participants included in the trial, and the thickness of lines between the interventions relates to the number of studies.

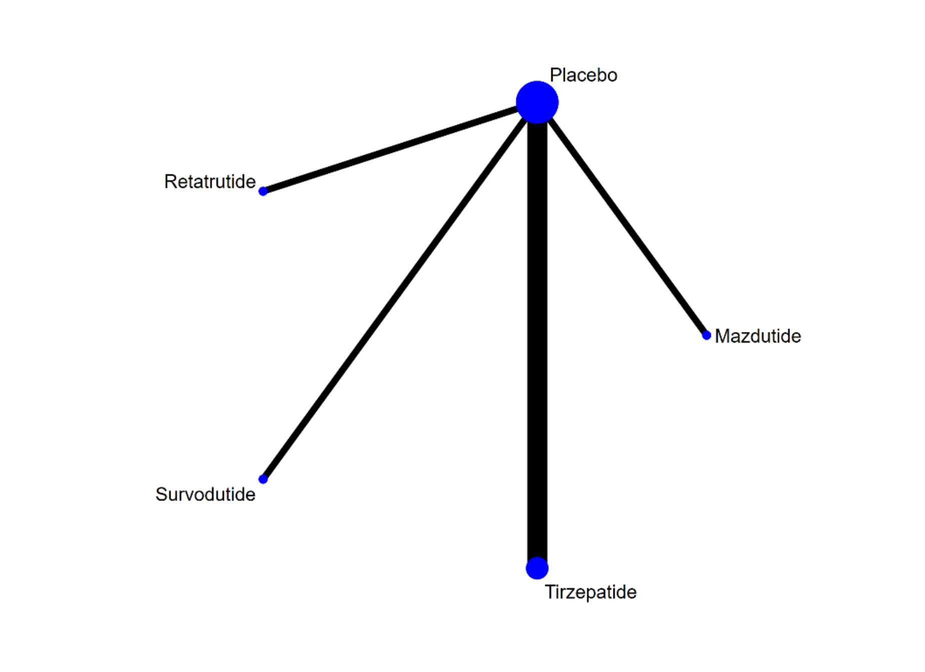

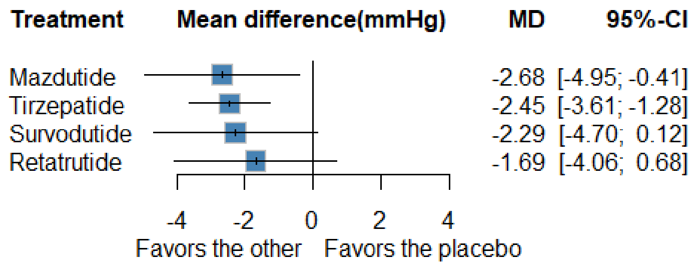

Figure S8.7: Network map of the effect on systolic blood pressure (patients without T2D), and forest plot of network effect sizes for comparison with placebo. The size of the nodes was proportional to the number of participants included in the trial, and the thickness of lines between the interventions relates to the number of studies.

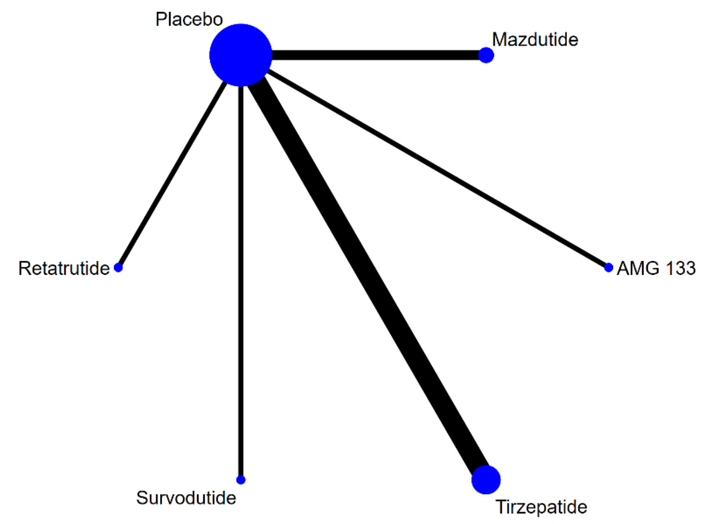

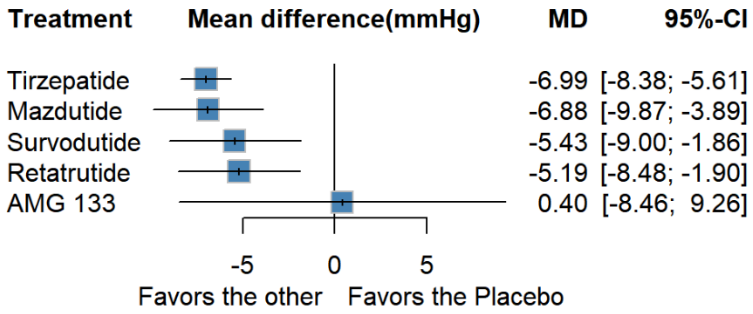

Figure S8.8: Network map of the effect on diastolic blood pressure (patients without T2D), and forest plot of network effect sizes for comparison with placebo. The size of the nodes was proportional to the number of participants included in the trial, and the thickness of lines between the interventions relates to the number of studies.

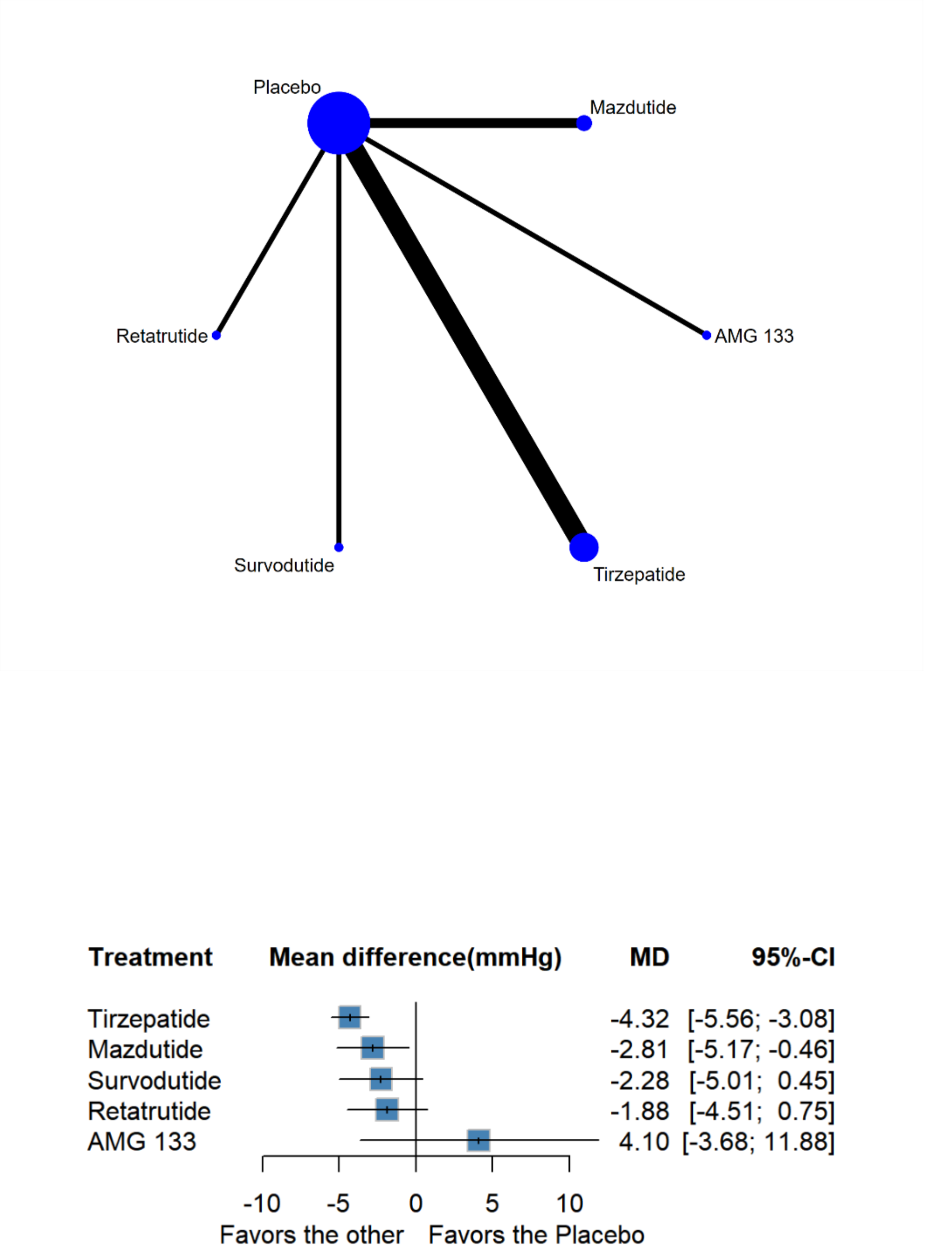

Figure S8.9: Network map of the effect on percent change of body weight (patients with T2D), and forest plot of network effect sizes for comparison with placebo. The size of the nodes was proportional to the number of participants included in the trial, and the thickness of lines between the interventions relates to the number of studies.

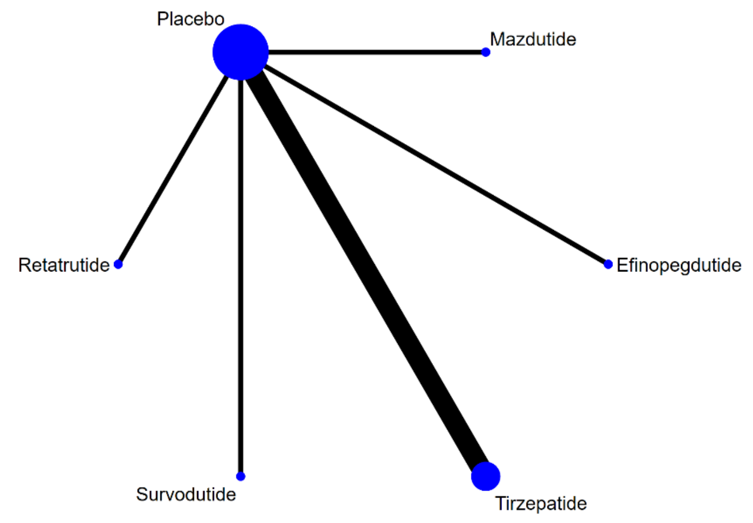

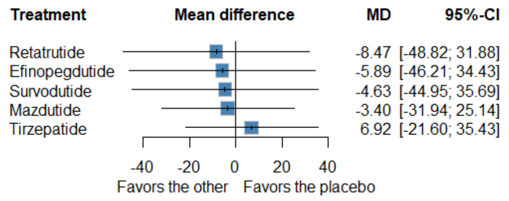

Figure S8.10: Network map of the effect on percent change of body weight (patients without T2D), and forest plot of network effect sizes for comparison with placebo. The size of the nodes was proportional to the number of participants included in the trial, and the thickness of lines between the interventions relates to the number of studies.

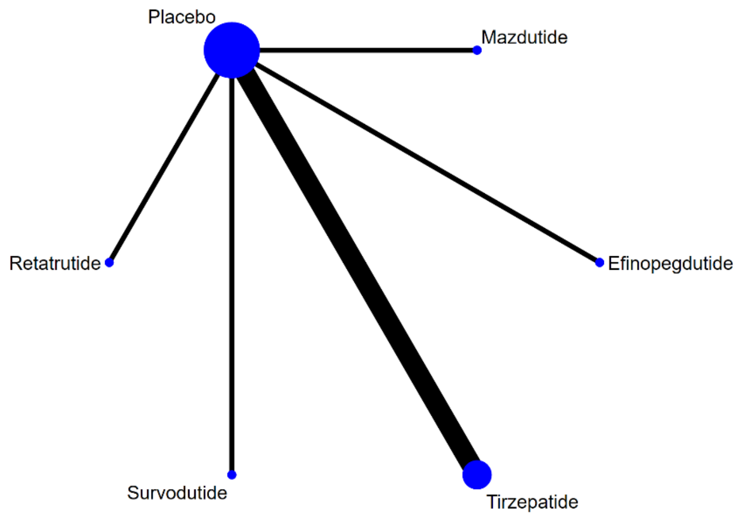

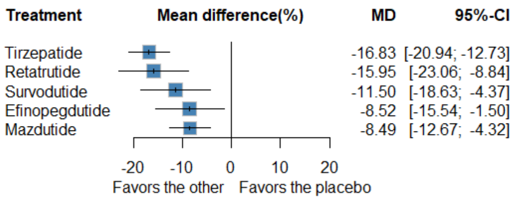

Appendix 9: Treatment ranking (P-scores) for the effects of Subgroup analysis of multi-receptor drugs on patients with or without T2D

Body Weight (T2D)		Body Weight (NT2D)		Weight Loss ≥5% (T2D)	
treatment	p-score	treatment	p-score	treatment	p-score
Tirzepatide	0.84	Tirzepatide	0.90	Tirzepatide	0.83
Survodutide	0.73	Retatrutide	0.89	Survodutide	0.74
Efinopegdutide	0.60	Efinopegdutide	0.46	Retatrutide	0.61
Retatrutide	0.56	Survodutide	0.44	Efinopegdutide	0.46
Mazdutide	0.24	Mazdutide	0.31	Mazdutide	0.37
Placebo	0.03	Placebo	0.00	Placebo	0.00
Weight Loss ≥5% (NT2D)		Systolic Blood Pressure (T2D)		Systolic Blood Pressure (NT2D)	
Mazdutide	0.83	Tirzepatide	0.67	Tirzepatide	0.82
Tirzepatide	0.71	Survodutide	0.66	Mazdutide	0.78
Retatrutide	0.62	Mazdutide	0.66	Survodutide	0.58
Survodutide	0.43	Retatrutide	0.50	Retatrutide	0.55
Efinopegdutide	0.40	Placebo	0.02	AMG133	0.16
Placebo	0.01			Placebo	0.11
Diastolic Blood Pressure (T2D)		Diastolic Blood Pressure (NT2D)		Body Weight (%: NT2D)	
Mazdutide	0.72	Tirzepatide	0.94	Tirzepatide	0.89
Tirzepatide	0.67	Mazdutide	0.68	Retatrutide	0.82
Survodutide	0.62	Survodutide	0.59	Survodutide	0.56
Retatrutide	0.46	Retatrutide	0.52	Efinopegdutide	0.37
Placebo	0.03	Placebo	0.20	Mazdutide	0.36
		AMG 133	0.07	Placebo	0.00

Appendix 10: League Table of Summary Estimates for Subgroup analysis of multi-receptor drugs on patients with or without T2D

Table S10.1a: Body Weight (with T2D)

The columns represent the comparison of the row drug class to the column drug class. The rows represent the comparison of the row drug class to the column drug class. The effect estimates are expressed as mean difference and 95% confidence interval. Mean difference <0 favors the drug in the column, Mean difference >0 favors the drug in the row.

**Tirzepatide**					
-0.86<br>(-5.50 to 3.77)	**Survodutide**				
-2.21<br>(-8.29 to 3.86)	-1.35<br>(-8.11 to 5.41)	**Efinopegdutide**			
-2.59<br>(-8.82 to 3.64)	-1.73<br>(-8.63 to 5.17)	-0.38<br>(-8.32 to 7.56)	**Retatrutide**		
-6.28<br>(-10.95 to -1.61)	-5.42<br>(-10.95 to 0.11)	-4.07<br>(-10.85 to 2.72)	-3.69<br>(-10.61 to 3.24)	**Mazdutide**	
-8.77<br>(-11.29 to -6.25)	-7.91<br>(-11.80 to -4.02)	-6.56<br>(-12.09 to -1.03)	-6.18<br>(-11.88 to -0.48)	-2.49<br>(-6.43 to 1.44)	**Placebo**

Table S10.1b: Body Weight (without T2D)

The columns represent the comparison of the row drug class to the column drug class. The rows represent the comparison of the row drug class to the column drug class. The effect estimates are expressed as mean difference and 95% confidence interval. Mean difference <0 favors the drug in the column, Mean difference >0 favors the drug in the row.

**Tirzepatide**					
-0.06<br>(-7.20 to 7.08)	**Retatrutide**				
-8.19<br>(-15.23 to -1.14)	-8.13<br>(-17.13 to 0.87)	**Efinopegdutide**			
-8.61<br>(-14.11 to -3.11)	-8.55<br>(-16.40 to -0.70)	-0.42<br>(-8.18 to 7.34)	**Survodutide**		
-10.51<br>(-15.35 to -5.68)	-10.45<br>(-17.85 to -3.06)	-2.32<br>(-9.63 to 4.98)	-1.90<br>(-7.73 to 3.92)	**Mazdutide**	
-17.75<br>(-20.88 to -14.61)	-17.69<br>(-24.11 to -11.27)	-9.56<br>(-15.87 to -3.25)	-9.14<br>(-13.66 to -4.62)	-7.24<br>(-10.92 to -3.55)	**Placebo**

Table S10.2a: Weight Loss (≥5%) (with T2D)

The columns represent the comparison of the row drug class to the column drug class. The rows represent the comparison of the row drug class to the column drug class. The effect estimates are expressed as odds ratio and 95% confidence interval. Odds ratio <1 favors the drug in the row, Odds ratio >1 favors the drug in the column.

**Tirzepatide**					
1.09<br>(0.32 to 3.70)	**Survodutide**				
1.43<br>(0.53 to 3.85)	1.32<br>(0.30 to 5.56)	**Retatrutide**			
1.96<br>(0.65 to 5.88)	1.82<br>(0.38 to 8.33)	1.37<br>(0.35 to 5.56)	**Efinopegdutide**		
2.44<br>(0.76 to 7.69)	2.22<br>(0.45 to 11.11)	1.69<br>(0.41 to 7.14)	1.23<br>(0.27 to 5.54)	**Mazdutide**	
14.5<br>(9.66 to 21.98)	13.33<br>(4.18 to 42.48)	10.14<br>(4.12 to 24.93)	7.38<br>(2.62 to 20.78)	6.00<br>(2.01 to 17.87)	**Placebo**

Table S10.2a: Weight Loss (≥5%) (without T2D)

The columns represent the comparison of the row drug class to the column drug class. The rows represent the comparison of the row drug class to the column drug class. The effect estimates are expressed as odds ratio and 95% confidence interval. Odds ratio <1 favors the drug in the row, Odds ratio >1 favors the drug in the column.

**Mazdutide**					
2.00<br>(0.15 to 26.10)	**Tirzepatide**				
2.58<br>(0.11 to 60.62)	1.30<br>(0.12 to 14.29)	**Retatrutide**			
5.75<br>(0.24 to 135.90)	2.86<br>(0.27 to 33.33)	2.23<br>(0.11 to 44.52)	**Survodutide**		
6.67<br>(0.27 to 100)	3.33<br>(0.30 to 33.33)	2.56<br>(0.12 to 50.00)	1.14<br>(0.06 to 25.00)	**Efinopegdutide**	
54.99<br>(5.27 to 573.24)	27.48<br>(9.62 to 78.51)	21.29<br>(2.57 to 176.18)	9.56<br>(1.14 to 79.82)	8.36<br>(0.97 to 72.27)	**Placebo**

Table S10.3a: Systolic Blood Pressure (with T2D)
The columns represent the comparison of the row drug class to the column drug class. The rows represent the comparison of the row drug class to the column drug class. The effect estimates are expressed as mean difference and 95% confidence interval. Mean difference <0 favors the drug in the column, Mean difference >0 favors the drug in the row.

**Survodutide**				
-0.01<br>(-5.24 to 5.22)	**Tirzepatide**			
-0.02<br>(-6.42 to 6.38)	-0.01<br>(-5.35 to 5.34)	**Mazdutide**		
-1.35<br>(- 8.32 to 5.62)	-1.34<br>(-7.35 to 4.68)	-1.33<br>(-8.39 to 5.73)	**Retatrutide**	
-5.81<br>(-10.27 to -1.35)	-5.80<br>(-8.53 to -3.06)	-5.79<br>(-10.38 to -1.20)	-4.46<br>(-9.82 to 0.90)	**Placebo**

Table S10.3b: Systolic Blood Pressure (without T2D)
The columns represent the comparison of the row drug class to the column drug class. The rows represent the comparison of the row drug class to the column drug class. The effect estimates are expressed as mean difference and 95% confidence interval. Mean difference <0 favors the drug in the column, Mean difference >0 favors the drug in the row.

**Tirzepatide**					
-0.12<br>(-3.41 to 3.18)	**Mazdutide**				
-1.56<br>(-5.39 to 2.26)	-1.45<br>(-6.11 to 3.21)	**Survodutide**			
-1.80<br>(-5.38 to 1.77)	-1.69<br>(-6.14 to 2.76)	-0.24<br>(-5.10 to 4.62)	**Retatrutide**		
-6.99<br>(-8.38 to -5.61)	-6.88<br>(-9.87 to -3.89)	-5.43<br>(-9.00 to -1.86)	-5.19<br>(-8.48 to -1.90)	**Placebo**	
-7.39<br>(-16.36 to 1.57)	-7.28<br>(-16.63 to 2.07)	-5.83<br>(-15.38 to 3.72)	-5.59<br>(-15.04 to 3.86)	-0.40<br>(-9.26 to 8.46)	**AMG133**

Table S10.4a: Diastolic Blood Pressure (with T2D)

The columns represent the comparison of the row drug class to the column drug class. The rows represent the comparison of the row drug class to the column drug class. The effect estimates are expressed as mean difference and 95% confidence interval. Mean difference <0 favors the drug in the column, Mean difference >0 favors the drug in the row.

**Mazdutide**				
-0.23<br>(-2.79 to 2.32)	**Tirzepatide**			
-0.39<br>(-3.70 to 2.92)	-0.16<br>(-2.84 to 2.52)	**Survodutide**		
-0.99<br>(-4.27 to 2.29)	-0.76<br>(-3.40 to 1.88)	-0.60<br>(-3.98 to 2.78)	**Retatrutide**	
-2.68<br>(-4.95 to -0.41)	-2.45<br>(-3.61 to -1.28)	-2.29<br>(-4.70 to 0.12)	-1.69<br>(-4.06 to 0.68)	**Placebo**

Table S10.4a: Diastolic Blood Pressure (without T2D)

The columns represent the comparison of the row drug class to the column drug class. The rows represent the comparison of the row drug class to the column drug class. The effect estimates are expressed as mean difference and 95% confidence interval. Mean difference <0 favors the drug in the column, Mean difference >0 favors the drug in the row.

**Tirzepatide**					
-1.50<br>(-4.17 to 1.16)	**Mazdutide**				
-2.04<br>(-5.03 to 0.96)	-0.53<br>(-4.14 to 3.07)	**Survodutide**			
-2.44<br>(-5.35 to 0.47)	-0.93<br>(-4.47 to 2.60)	-0.40<br>(-4.19 to 3.39)	**Retatrutide**		
-4.32<br>(-5.56 to -3.08)	-2.81<br>(-5.17 to -0.46)	-2.28<br>(-5.01 to 0.45)	-1.88<br>(-4.51 to 0.75)	**Placebo**	
-8.42<br>(-16.30 to 0.54)	-6.91<br>(-15.04 to 1.22)	-6.38<br>(-14.62 to 1.86)	-5.98<br>(-14.19 to 2.23)	-4.10<br>(-11.88 to 3.68)	**AMG 133**

Appendix 11: Comprehensive comparisons of different multi-receptor drugs at various doses

Figure S11.1: HbA_1c

Reference treatment: Placebo

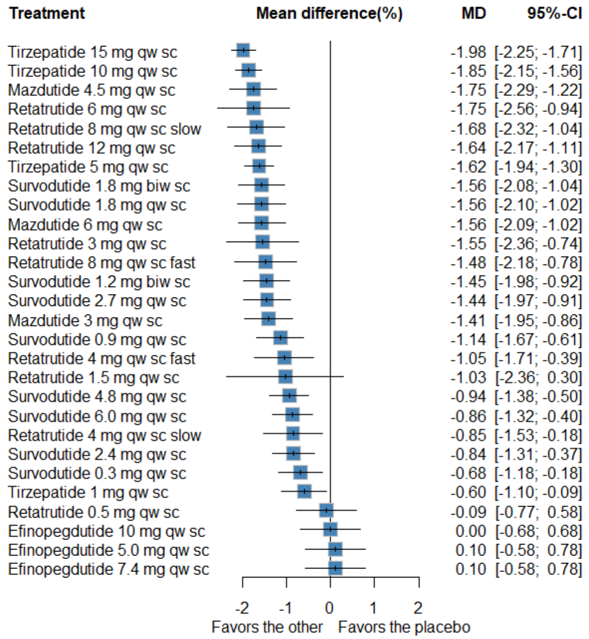

Figure S11.2: FPG
Reference treatment: Placebo

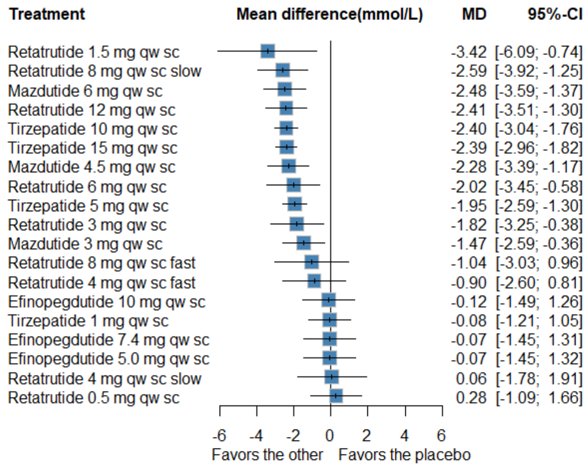

Figure S11.3: Body Weight
Reference treatment: Placebo

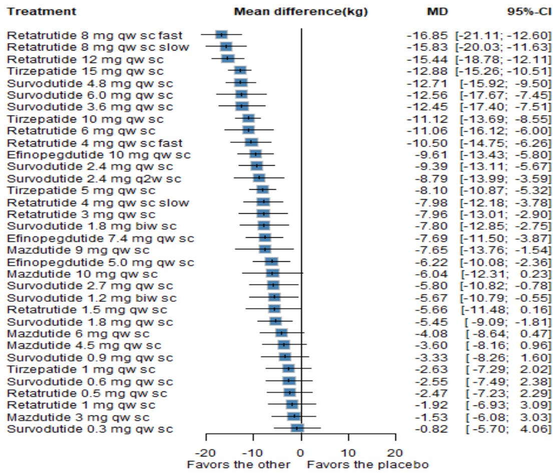

Figure S11.4: Weight Loss (≥5%)
Reference treatment: Placebo

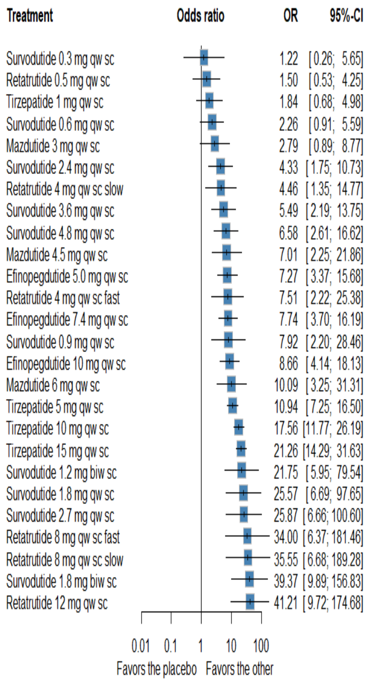

Figure S11.5: Waist Circumference

Reference treatment: Placebo

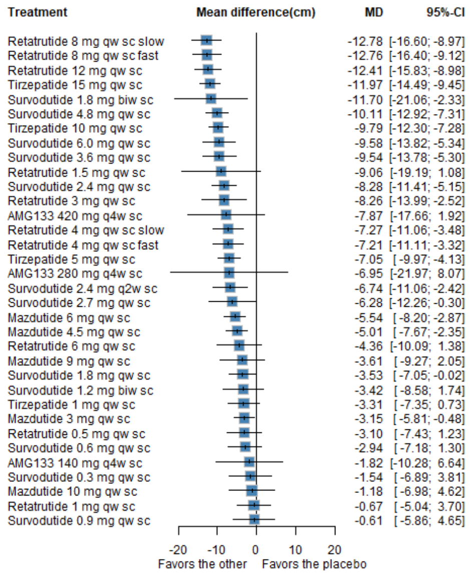

Figure S11.6: Systolic Blood Pressure
Reference treatment: Placebo

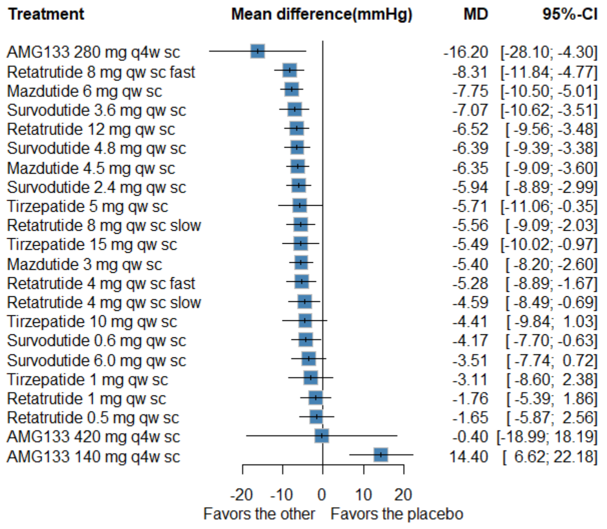

Figure S11.7: Diastolic Blood Pressure

Reference treatment: Placebo

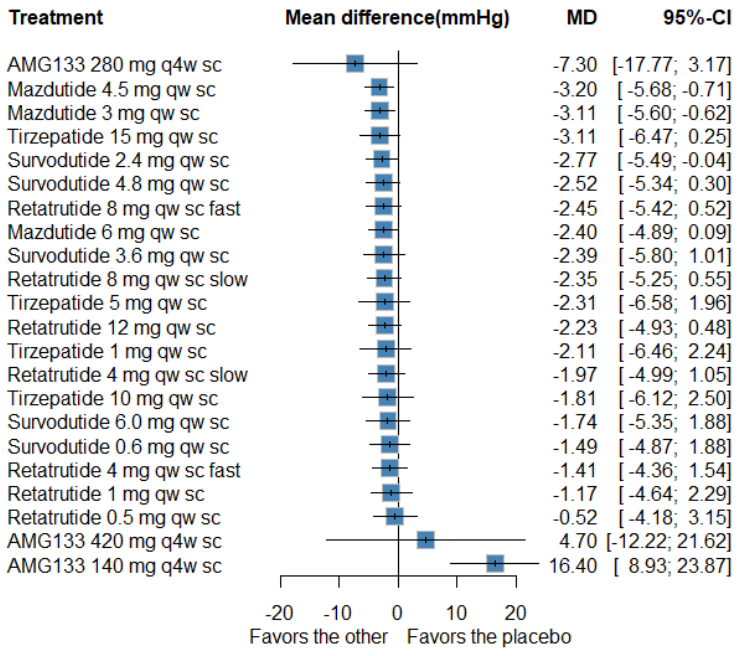

Figure S11.8: Adverse Effect

Reference treatment: Placebo

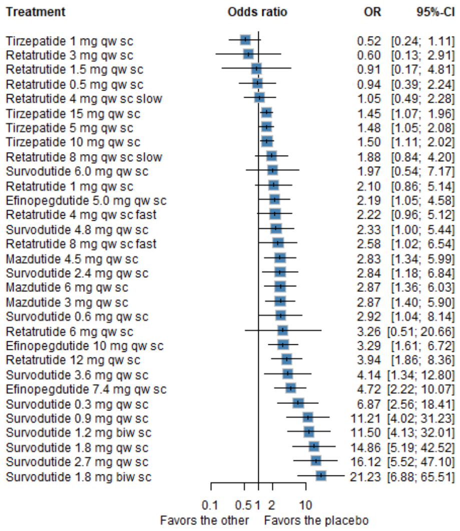

Figure S11.9: Serious Adverse Effect
Reference treatment: Placebo

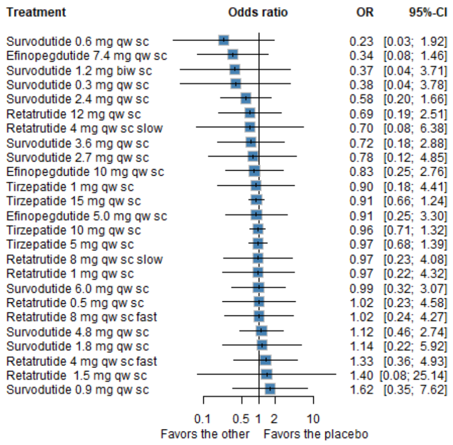

Figure S11.10: BMI
Reference treatment: Placebo

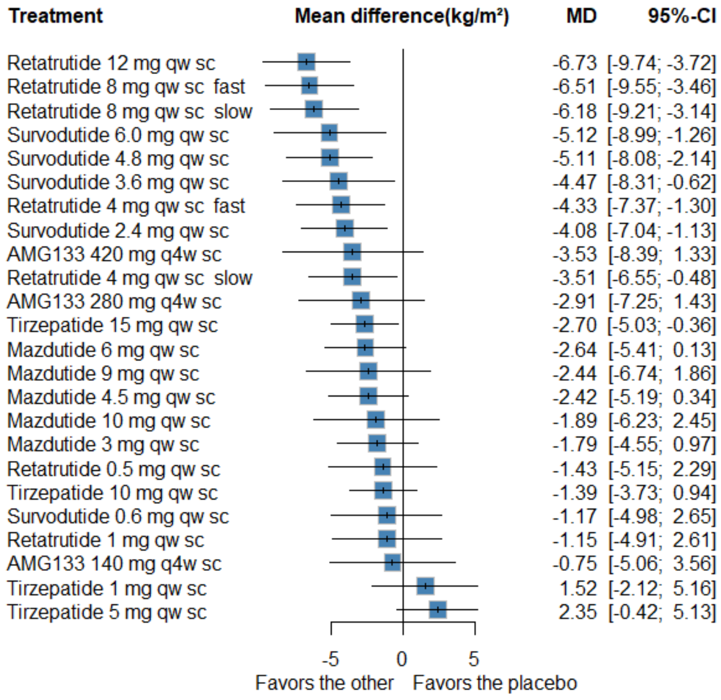

Appendix 12: Study details and participant baseline characteristics of included arms in RCT

Table S12: Study details and participant baseline characteristics of included arms in RCTs

Study	Trial registration no.	Study duration weeks	Blinding status	Design	Number of participants	Randomized treatments	Dose and frequency	Age years	Male %	Diabetes duration, years	HbA_{1c} %	FPG mmol/L	BMI	SBP mmHg	DBP mmHg
Matthias2024(1)	NCT04153929	16 weeks	Double-blind	RCT	361	Placebo: 59		57.5 ± 10.5	52.5%	7.9 ± 5.6	8.15 ± 0.85		33.4 ± 5.9		
						Survodutide: 50	0.3 mg qw sc	56.1 ± 10.2	52.0%	6.1 ± 4.7	8.09 ± 0.76		33.8 ± 6.1		
						Survodutide: 50	0.9 mg qw sc	58.2 ± 9.6	56.0%	7.7 ± 7.3	7.89 ± 0.80		34.9 ± 5.2		
						Survodutide: 52	1.8 mg qw sc	55.3 ± 10.3	51.9%	7.0 ± 5.6	8.14 ± 0.86		33.6 ± 5.8		
						Survodutide: 50	2.7 mg qw sc	59.6 ± 8.5	66.0%	7.9 ± 5.7	8.18 ± 0.97		34.0 ± 6.8		
						Survodutide: 51	1.2 mg biw sc	58.3 ± 8.8	52.9%	8.8 ± 7.1	8.11 ± 0.94		33.0 ± 5.0		
						Survodutide: 49	1.8 mg biw sc	57.7 ± 9.4	55.1%	7.4 ± 5.3	7.97 ± 0.71		34.9 ± 7.0		
Heise2022(2)	NCT03951753	28 weeks	Double-blind	RCT	73	Placebo:28		60.4 ± 7.6	75.0%	10.95 ± 6.78	7.90 ± 0.51	7.0 ± 1.3	31.28±5.01	80.3 ± 7.0	135.9 ± 14.5
						Tirzepatide: 45	15 mg qw sc	61.1 ± 7.1	69.0%	10.24 ± 5.80	7.83 ± 0.72	7.7 ± 1.7	32.24±3.96	81.3 ± 7.6	137.2 ± 13.0
Zhang2024(3)	NCT04965506	20 weeks	Double-blind	RCT	200	Placebo:51		52.6 ± 11.1	54.9%	3.8 ± 4.9	8.16 ± 0.91	9.2 ± 2.2	27.5 ± 3.5	82.0 ± 6.8	128.7 ± 12.8
						Mazdutide:51	3 mg qw sc	52.5 ± 13.4	51.0%	3.6 ± 3.0	8.11 ± 0.84	9.3 ± 2.5	28.0 ± 4.8	82.3 ± 8.6	128.5 ± 13.1
						Mazdutide:49	4.5 mg qw sc	55. ± 10.7	65.3%	4.4 ± 3.9	8.11 ± 0.91	9.3 ± 1.9	27.3 ± 3.8	82.6 ± 7.9	130.5 ± 11.2
						Mazdutide:49	6 mg qw sc	54.4 ± 9.8	73.5%	3.3 ± 3.0	7.94 ± 0.93	9.6 ± 2.2	27.2 ± 3.4	82.6 ± 8.1	128.9 ± 11.7
Jiang2022(4)	NCT04466904	12 weeks	double-blind	RCT	42	Placebo :12		50.2 ± 8.5	50.0%	3.1 ± 2.4	8.3 ± 0.7	11.2 ± 2.6	26.5 ± 2.7	80.5 ± 7.8	121.3 ± 12.4
						Mazdutide: 8	3 mg qw sc	58.6 ± 5.3	75.0%	2.1 ± 0.9	8.9 ± 0.7	11.1 ± 1.9	24.1 ± 1.4	82.3 ± 12.2	129.9 ± 14.3

						Mazdutide: 8	4.5 mg qw sc	47.9 ± 8.9	37.5%	4.7 ± 2.4	8.8 ± 1.0	10.7 ± 1.8	25.7±3.4	81.9 ± 7.6	120.8 ± 11.0
						Mazdutide: 8	6 mg qw sc	54.6 ± 10.6	62.5%	6.0 ± 5.0	8.5 ± 1.1	11.7 ± 2.2	26.0±2.2	75.3 ± 9.0	122.1 ± 15.9
Di Prospero2021(5)	NCT03586830	12 weeks	Double-blind	RCT	195	Placebo: 49		57.4 ± 9.1	38.8%	7.6 ± 6.4	7.5 ± 0.9	8.4 ± 2.1	39.5 ± 4.4	77.7 ± 6.4	126.0 ± 12.1
						Efinopegdutide: 48	5.0 mg qw sc	55.1 ± 9.6	33.3%	6.4 ± 4.3	7.4 ± 0.8	8.3 ± 2.0	40.4 ± 4.0	77.5 ± 9.7	127.3 ± 14.6
						Efinopegdutide: 49	7.4 mg qw sc	57.8 ± 9.0	42.9%	8.7 ± 6.0	7.5 ± 0.8	8.7 ± 2.2	40.4 ± 4.2	77.9 ± 7.3	130.3 ± 10.4
						Efinopegdutide: 49	10.0 mg qw sc	56.2 ± 8.6	42.9%	8.2 ± 7.9	7.8 ± 1.0	8.9 ± 3.1	41.0 ± 4.0	77.0 ± 8.6	126.3 ± 13.9
Dahl2022(6)	NCT04039503	40 weeks	Double-blind	RCT	475	Placebo: 120		60 ± 10	55.0%	12.9 ± 7.4	8.37± 0.84	9.1 ± 2.5	33.2 ± 6.3	83 ± 10	140 ± 15
						Tirzepatide:116	5 mg qw sc	62 ± 10	53.0%	14.1 ± 8.1	8.30 ± 0.88	9.1 ± 3.0	33.6 ± 5.9	79 ± 12	137 ± 16
						Tirzepatide: 119	10 mg qw sc	60 ± 10	61.0%	12.6 ± 6.2	8.36± 0.83	9.0 ± 2.9	33.4 ± 6.2	81 ± 10	138 ± 15
						Tirzepatide: 120	15 mg qw sc	61 ± 10	54.0%	13.7 ± 7.5	8.23± 0.86	8.9 ± 3.0	33.4 ± 5.9	80 ± 11	137 ± 16
Wadden2023(7)	NCT04657016	72 weeks	Double-blind	RCT	579	Placebo: 292		45.7 ± 11.8	37.3%						
						Tirzepatide: 287	10 mg/15 mg qw sc	45.4 ± 12.6	36.9%						
Urva2022(8)	NCT04143802	12 weeks	Double-blind	RCT	67	Placebo: 15		58.8 ± 6.4	20.0%	9.2 ± 6.0	8.83 ± 1.06		32·3 ± 6·2		
						Retatrutide: 9	0.5 mg qw sc	59.2 ± 6.6	44.0%	10.7 ± 5.2	8.07 ± 0.74		33·3 ± 6·3		
						Retatrutide: 9	1.5 mg qw sc	56.8 ± 5.7	33.0%	9.0 ± 5.8	8.87 ± 0.79		32·4 ± 6·1		
						Retatrutide: 11	3 mg qw sc	56.8 ± 8.0	55.0%	10.2 ± 4.6	8.65 ± 0.98		31·7 ± 5·1		
						Retatrutide: 11	6 mg qw sc	55.8 ± 10.7	64.0%	12.9 ± 7.7	9.05 ± 0.81		33·7 ± 3·9		
						Retatrutide: 12	12 mg qw sc	61.5 ± 6.3	67.0%	10.0 ± 5.4	8.45 ± 0.92		30·5 ± 3·6		
Rosenstock2023(9)	NCT04867785	36 weeks	Double-blind	RCT	235	Placebo:45		57.6 ± 10.8	49.0%	8.7 ± 8.3	8.4 ± 1.1	10.2 ± 3.4	33.8 ± 4.9	78.6 ± 9.8	131.9 ± 15.0
						Retatrutide: 47	0.5 mg qw sc	57.2 ± 9.7	51.0%	8.8 ± 6.7	8.3 ± 1.2	9.7 ± 3.7	34.7 ± 5.6	79.9 ± 8.0	132.0 ± 11.6

						Retatrutide: 23	4 mg qw sc slow	57.7 ± 8.1	65.0%	8.1 ± 6.6	8.1 ± 0.9	9.5 ± 2.7	36.3 ± 7.4	82.4 ± 7.3	135.4 ± 9.8
						Retatrutide: 24	4 mg qw sc fast	57.6 ± 10.0	50.0%	10.5 ± 7.6	8.2 ± 1.2	9.7 ± 2.9	34.0 ± 6.5	77.4 ± 9.9	125.8 ± 12.8
						Retatrutide :26	8 mg qw sc slow	57.0 ± 7.4	38.0%	7.2 ± 6.4	8.3 ± 1.1	9.9 ± 2.8	35.0 ± 6.4	78.2 ± 9.6	131.0 ± 11.9
						Retatrutide: 24	8 mg qw sc fast	53.8 ± 9.0	38.0%	6.0 ± 5.8	8.2 ± 1.3	8.5 ± 2.1	34.1 ± 5.9	82.6 ± 7.2	131.6 ± 11.6
						Retatrutide: 46	12 mg qw sc	54.4 ± 9.7	43.0%	7.9 ± 6.9	8.3 ± 1.1	9.6 ± 3.1	35.5 ± 6.9	78.7 ± 8.4	124.7 ± 13.7
Rosenstock2021(10)	NCT03954834	40 weeks	Double-blind	RCT	478	Placebo :115		53.6 ± 12.8	49.0%	4.5 ± 5.9	8.05 ± 0.80	8.6 ± 2.2	32.2 ± 7.0	79.7 ± 9.3	127.8 ± 14.1
						Tirzepatide:121	5 mg qw sc	54.1 ± 11.9	46.0%	4.6± 5.1	7.97 ± 0.84	8.5 ± 2.1	32.2 ± 7.6	79.9 ± 9.0	128.2 ± 15.7
						Tirzepatide: 121	10 mg qw sc	55.8 ± 10.4	60.0%	4.9 ± 5.6	7.90 ± 0.7	8.5 ± 2.3	31.5 ± 5.5	78.7 ± 8.2	127.8 ± 12.6
						Tirzepatide: 121	15 mg qw sc	52.9 ± 12.3	52.0%	4.8 ± 5.0	7.85 ± 1.02	8.5 ± 2.2	31.7 ± 6.1	79.2 ± 8.8	126.8 ± 13.8
Frias2018(11)	NCT03131687	26 weeks	Double-blind	RCT	263	Placebo:51		56.6 ± 8.9	57.0%	8.6 ± 7.0	8.0 ± 0.9	9.1 ± 2.3	32.4 ± 6.0	75.1 ± 8.2	124.8 ± 13.0
						Tirzepatide: 52	1mg qw sc	57.4 ± 8.9	56.0%	7.8 ± 5.4	8.2 ± 0.9	8.9 ± 2.3	32.9 ± 6.1	75.2 ± 8.2	126.8 ± 13.0
						Tirzepatide: 55	5 mg qw sc	57.9 ± 8.2	62.0%	8.9 ± 5.7	8.2 ± 1.0	9.4 ± 2.5	32.9 ± 5.7	76.6 ± 8.2	127.8 ± 13.0
						Tirzepatide: 51	10 mg qw sc	56.5 ± 9.9	59.0%	7.9 ± 5.8	8.2 ± 1.1	9.5 ± 2.8	32.6 ± 5.8	76.2 ± 8.2	125.8± 12.8
						Tirzepatide: 53	15 mg qw sc	56.0 ± 7.6	42.0%	8.5 ± 6.1	8.1 ± 1.1	9.2 ± 2.7	32.2 ± 6.2	77.1 ± 8.3	126.0 ± 13.0
Garvey2023(12)	NCT04657003	72 weeks	Double-blind	RCT	938	Placebo:315		54.7 ± 10.5	50.0%	8.8 ± 6.2	7.89 ± 0.84	8.8 ± 2.6	36.6±7.3	79.4 ± 8.4	131.0 ± 11.9
						Tirzepatide: 312	10 mg qw sc	54.3 ± 10.7	49.0%	8.8 ± 6.9	8.00 ± 0.84	8.8 ± 2.4	36.0 ± 6.4	80.2 ± 8.1	130.6 ± 12.2
						Tirzepatide: 311	15 mg qw sc	53.6 ± 10.6	49.0%	8.0 ± 6.4	8.07 ± 0.99	9.0 ± 2.7	35.7 ± 6.1	79.7 ± 8.7	130.0 ± 12.3
Aronne2024(13)	NCT04660643	52 weeks	Open-label	RCT	770	Placebo: 335		48.0 ± 12.0	29.3%		5.04 ± 0.31	4.7 ± 0.4	30.3 ± 6.0	76.0 ± 9.0	115.0 ± 12.0
						Tirzepatide: 335	10 mg/15mg qw sc	49.0 ± 13.0	29.6%		5.07 ± 0.30	4.7 ± 0.4	30.7 ± 6.8	75.0 ± 9.0	115.0 ± 13.0

Alba2021(14)	NCT03486392	26 weeks	Double-blind	RCT	355	Placebo：60		46.9 ± 11.8	20.0%	5.50 ± 0.40	5.5 ± 0.5	40.4 ± 4.1	79.9 ± 9.1	128.2 ± 11.8
						Efinopegdutide:59	5.0 mg qw sc	47.3 ± 11.2	20.3%	5.50 ± 0.40	5.4 ± 0.7	40.3 ± 4.3	79.8 ± 11.1	126.5 ± 12.2
						Efinopegdutide:118	7.4 mg qw sc	46.2 ± 11.7	27.1%	5.50 ± 0.40	5.5 ± 0.7	40.0 ± 4.0	80.3 ± 9.7	127.4 ± 13.2
						Efinopegdutide:118	10 mg qw sc	46.2 ± 12.2	27.1%	5.50 ± 0.40	5.5 ± 0.6	40.5 ± 3.9	79.3 ± 9.1	125.4 ± 12.5
Jastreboff2023(15)	NCT04881760	48 weeks	Double-blind	RCT	338	Placebo: 70		48.0 ± 12.5	51.0%	5.50 ± 0.40	5.3 ± 0.5	37.3±5.9	83.5 ± 9.5	126.2 ± 12.6
						Retatrutide: 69	1 mg qw sc	50.6 ± 13.3	52.0%	5.50 ± 0.40	5.2 ± 0.5	37.5±5.9	79.7 ± 9.2	126.1 ± 14.6
						Retatrutide: 33	4 mg qw sc slow	50.8 ± 11.9	52.0%	5.60 ± 0.40	5.2 ± 0.5	37.3±5.9	80.9 ± 8.5	127.2 ± 12.4
						Retatrutide: 34	4 mg qw sc fast	46.8 ± 14.1	53.0%	5.50 ± 0.40	5.2 ± 0.4	37.4±4.7	81.0 ± 8.7	126.5 ± 13.3
						Retatrutide: 35	8 mg qw sc slow	46.1 ± 13.5	51.0%	5.50 ± 0.40	5.0 ± 0.6	37.4±6.0	82.1 ± 9.1	126.3 ± 12.8
						Retatrutide: 35	8 mg qw sc fast	48.7 ± 11.1	51.0%	5.50 ± 0.40	5.2 ± 0.7	37.0±5.5	80.4 ± 9.3	122.3 ± 12.2
						Retatrutide: 62	12 mg qw sc	45.8 ± 12.2	52.0%	5.50 ± 0.40	5.2 ± 0.5	37.4±6.0	78.2 ± 11.3	118.7 ± 15.5
Arun2024(16)	NCT04771273	48 weeks	Double-blind	RCT	293	Placebo: 74		53.0 ± 11.5	41.0%	7.08 ± 0.87		35.30±5.05	81.2 ± 8.4	129.4 ± 12.5
						Survodutide: 73	2.4 mg qw sc	49.6 ± 13.7	51.0%	6.90 ± 1.12		35.00±6.97	80.4 ± 8.2	128.8 ± 14.8
						Survodutide: 72	4.8 mg qw sc	50.2 ± 12.9	53.0%	6.90 ± 1.06		37.42±6.84	81.8 ± 9.4	132.4 ± 14.1
						Survodutide: 74	6.0 mg qw sc	50.4 ± 13.1	45.0%	6.92 ± 0.91		35.49±6.44	79.4 ± 8.1	127.0 ± 14.8
Zhao2023(17)	NCT05024032	52 weeks	Double-blind	RCT	210	Placebo:69		37.8 ± 10.2	52.2%	5.65 ± 0.29	5.2 ± 0.4	32.4 ± 3.6	83 ± 8	121.0 ± 13.0
						Tirzepatide: 70	10 mg qw sc	34.7 ± 7.2	50.0%	5.60 ± 0.35	5.1 ± 0.5	32.6 ± 4.1	82 ± 9	120.0 ± 12.0
						Tirzepatide: 71	15 mg qw sc	35.8 ± 9.3	50.7%	5.57 ± 0.32	5.1 ± 0.5	32.0 ± 3.7	82 ± 9	119.0 ± 12.0
Yazawa2023(18)	NCT04384081	16 weeks	Single-blind	RCT	36	Placebo:9		36.2 ± 8.5	100.0%	5.21 ± 0.20	5.49 ± 0.39	25.9 ± 2.0		
						Survodutide:9	1.8 mg qw sc	37.0 ± 8.0	100.0%	5.20 ± 0.25	5.34 ± 0.25	25.6 ± 1.9		

						Survodutide:9	4.8 mg qw sc	31.9 ± 5.8	100.0%	5.12 ± 0.38	5.45 ± 0.54	24.1 ± 0.8		
						Survodutide:9	2.4 mg q2w sc	31.6 ± 7.5	100.0%	5.12 ± 0.21	5.52 ± 0.29	25.1 ± 1.9		
Ji2023(19)	NCT04904913	24 weeks	Double-blind	RCT	248	Placebo:62		35.5 ± 7.1	51.6%	5.45 ± 0.35	5.2 ± 0.6	32.0 ± 4.2	80.9 ± 8.5	118.5 ± 12.3
						Mazdutide:62	3 mg qw sc	37.2 ± 10.7	43.5%	5.41 ± 0.27	5.1 ± 0.4	31.8 ± 3.9	80.9 ± 7.9	119.2 ± 12.0
						Mazdutide:63	4.5 mg qw sc	33.6 ± 10.0	41.3%	5.40 ± 0.32	5.1 ± 0.4	31.8 ± 4.7	79.4 ± 7.6	116.3 ± 11.4
						Mazdutide:61	6 mg qw sc	35.8 ± 9.2	55.7%	5.34 ± 0.35	5.1 ± 0.4	31.7 ± 4.0	81.3 ± 8.5	120.8 ± 9.6
Jastreboff2022(20)	NCT04184622	72 weeks	Double-blind	RCT	2539	Placebo:643		44.4 ± 12.5	32.2%	5.6 ± 0.38	5.3 ± 0.5	38.2±6.89	79.6 ± 7.95	122.9 ± 12.77
						Tirzepatide: 630	5 mg qw sc	45.6 ± 12.7	32.4%	5.6 ± 0.36	5.3 ± 0.5	37.4±6.63	79.3 ± 8.14	123.6 ± 12.45
						Tirzepatide: 636	10 mg qw sc	44.7 ± 12.4	32.9%	5.6 ± 0.37	5.3 ± 0.6	38.2±7.01	79.9 ± 8.32	123.8 ± 12.77
						Tirzepatide: 630	15 mg qw sc	44.9 ± 12.3	32.5%	5.6 ± 0.41	5.3 ± 0.6	38.1±6.69	79.3 ± 8.23	123.0 ± 12.94
Véniant2024(21)	NCT04478708	30 weeks	Double-blind	RCT	26	Placebo:6		45.7 ± 14.0	33.3%	5.50 ± 0.20		34.2 ± 3.7		
						AMG133:6	140 mg q4w sc	40.3 ± 16.6	83.3%	5.60 ± 0.48		34.1 ± 2.9		
						AMG133:6	280 mg q4w sc	44.5 ± 13.8	66.7%	5.57 ± 0.33		33.4 ± 3.6		
						AMG133:8	420 mg q4w sc	51.6 ± 12.8	12.5%	5.58 ± 0.27		32.5 ± 2.6		
Roux2024(22)	NCT04667377	46 weeks	Double-blind	RCT	384	Placebo:77		50.0 ± 13.5	31.0%	5.45 ± 0.36		37.6 ± 6.0	82.4 ± 8.6	127.5 ± 14.2
						Survodutide:77	0.6 mg qw sc	48.6 ± 12.6	34.0%	5.51 ± 0.43		37.8 ± 6.3	80.5 ± 7.5	125.0 ± 13.4
						Survodutide:78	2.4 mg qw sc	49.0 ± 13.1	31.0%	5.48 ± 0.46		37.6 ± 7.3	80.7 ± 7.4	125.4 ± 13.3
						Survodutide:76	3.6 mg qw sc	50.3 ± 11.8	33.0%	5.56 ± 0.38		37.0 ± 5.7	81.8 ± 8.1	127.4 ± 13.2
						Survodutide:76	4.8 mg qw sc	47.6 ± 13.5	50.0%	5.50 ± 0.42		37.8 ± 6.3	80.8 ± 7.6	122.6 ± 12.3
Ji2022(23)	NCT04440345	16 weeks	Open-label	RCT	24	Placebo:8		39.9 ± 11.7	37.5%			29.6 ± 3.3		

						Mazdutide: 8	9 mg qw sc	37.9 ± 9.7	25.0%				30.1 ± 3.8			
						Mazdutide: 8	10 mg qw sc	36.0 ± 9.1	25.0%				31.8 ± 5.1			
Ji2021(24)	NCT04440345	12 weeks	Open-label	RCT	36	Placebo:12		35.0 ± 7.4	33.3%		5.2 ± 0.3	5.5 ± 0.4	29.1 ± 3.7		78.0 ± 10.8	114.8 ± 9.5
						Mazdutide :8	3 mg qw sc	29.5 ± 15.6	37.5%		5.3 ± 0.6	5.5 ± 0.2	29.3 ± 2.2		79.3 ± 9.7	118.5 ± 10.5
						Mazdutide: 8	4.5 mg qw sc	31.0 ± 7.0	75.0%		5.2 ± 0.2	5.4 ± 0.3	32.4 ± 3.4		80.8 ± 7.4	122.0 ± 11.3
						Mazdutide: 8	6 mg qw sc	40.0 ± 21.1	62.5%		5.3 ± 0.3	5.1 ± 0.6	31.7 ± 2.0		81.3 ± 7.3	124.1 ± 6.9

Data are shown as mean ± SD.

Abbreviations: FPG, fasting plasma glucose; SC, subcutaneous injections; qw, once weekly; q4w: once four-weekly; biw: twice weekly; Systolic Blood pressure; DBP: diastolic blood pressure; RCT: Randomized Controlled Trial

Appendix 13: Definition of serious Adverse Events (SAEs).

Serious adverse events are defined as any untoward medical occurrence that, at any dose, meets the following criteria:

1. **Results in death**
2. **Is life-threatening**
   - The term "life-threatening" refers to an event in which the participant was at risk of death at the time of the event. It does not refer to an event that hypothetically might have caused death if it had been more severe.
3. **Requires inpatient hospitalization or prolongation of existing hospitalization**
   - In general, hospitalization signifies that the participant has been admitted to a hospital for observation and/or treatment that would not have been appropriate in a physician's office or outpatient setting.
   - Complications that occur during hospitalization are adverse events (AEs). If a complication prolongs hospitalization or fulfills any other serious criteria, the event is classified as serious.
   - Hospitalization for elective treatment of a pre-existing condition that did not worsen from baseline is not considered an AE.
4. **Results in persistent disability/incapacity**
   - Disability refers to a substantial disruption of a person's ability to conduct normal life functions.
   - This definition does not include relatively minor medical conditions, such as uncomplicated headache, nausea, vomiting, diarrhea, influenza, or accidental trauma (e.g., sprained ankle), which may interfere with everyday life functions but do not constitute a substantial disruption.
5. **Is a congenital anomaly/birth defect**
   - Abnormal pregnancy outcomes, including spontaneous abortion, fetal death, stillbirth, congenital anomalies, and ectopic pregnancy, are considered SAEs.
6. **Other situations**
   - Medical or scientific judgment should be exercised in deciding whether SAE reporting is appropriate in situations such as important medical events that may not be immediately life-threatening or result in death or hospitalization but may jeopardize the participant or may require medical or surgical intervention to prevent one of the other outcomes listed in the definition above.
   - These events should usually be considered serious.

Examples:

- Invasive or malignant cancers
- Intensive treatment in an emergency room or at home for allergic bronchospasm
- Blood dyscrasias or seizures that do not result in hospitalization
- Development of drug dependency or drug abuse

7. **Resulted in medical or surgical intervention to prevent life-threatening illness or injury or permanent impairment to a body structure or function**

Reference

1. Blüher M, Rosenstock J, Hoefler J, Manuel R, Hennige AM. Dose-Response Effects on Hba(1c) and Bodyweight Reduction of Survodutide, a Dual Glucagon/Glp-1 Receptor Agonist, Compared with Placebo and Open-Label Semaglutide in People with Type 2 Diabetes: A Randomised Clinical Trial. *Diabetologia* (2024) 67(3):470-82. Epub 2023/12/14. doi: 10.1007/s00125-023-06053-9.
2. Heise T, Mari A, DeVries JH, Urva S, Li J, Pratt EJ, et al. Effects of Subcutaneous Tirzepatide Versus Placebo or Semaglutide on Pancreatic Islet Function and Insulin Sensitivity in Adults with Type 2 Diabetes: A Multicentre, Randomised, Double-Blind, Parallel-Arm, Phase 1 Clinical Trial. *The lancet Diabetes & endocrinology* (2022) 10(6):418-29. Epub 2022/04/26. doi: 10.1016/s2213-8587(22)00085-7.
3. Zhang B, Cheng Z, Chen J, Zhang X, Liu D, Jiang H, et al. Efficacy and Safety of Mazdutide in Chinese Patients with Type 2 Diabetes: A Randomized, Double-Blind, Placebo-Controlled Phase 2 Trial. *Diabetes care* (2024) 47(1):160-8. Epub 2023/11/09. doi: 10.2337/dc23-1287.
4. Jiang H, Pang S, Zhang Y, Yu T, Liu M, Deng H, et al. A Phase 1b Randomised Controlled Trial of a Glucagon-Like Peptide-1 and Glucagon Receptor Dual Agonist Ibi362 (Ly3305677) in Chinese Patients with Type 2 Diabetes. *Nature communications* (2022) 13(1):3613. Epub 2022/06/25. doi: 10.1038/s41467-022-31328-x.
5. Di Prospero NA, Yee J, Frustaci ME, Samtani MN, Alba M, Fleck P. Efficacy and Safety of Glucagon-Like Peptide-1/Glucagon Receptor Co-Agonist Jnj-64565111 in Individuals with Type 2 Diabetes Mellitus and Obesity: A Randomized Dose-Ranging Study. *Clinical obesity* (2021) 11(2):e12433. Epub 2021/01/22. doi: 10.1111/cob.12433.
6. Dahl D, Onishi Y, Norwood P, Huh R, Bray R, Patel H, et al. Effect of Subcutaneous Tirzepatide Vs Placebo Added to Titrated Insulin Glargine on Glycemic Control in Patients with Type 2 Diabetes: The Surpass-5 Randomized Clinical Trial. *Jama* (2022) 327(6):534-45. Epub 2022/02/09. doi: 10.1001/jama.2022.0078.
7. Wadden TA, Chao AM, Machineni S, Kushner R, Ard J, Srivastava G, et al. Tirzepatide after Intensive Lifestyle Intervention in Adults with Overweight or Obesity: The Surmount-3 Phase 3 Trial. *Nature medicine* (2023) 29(11):2909-18. Epub 2023/10/16. doi: 10.1038/s41591-023-02597-w.
8. Urva S, Coskun T, Loh MT, Du Y, Thomas MK, Gurbuz S, et al. Ly3437943, a Novel Triple Gip, Glp-1, and Glucagon Receptor Agonist in People with Type 2 Diabetes: A Phase 1b, Multicentre, Double-Blind, Placebo-Controlled, Randomised, Multiple-Ascending Dose Trial. *Lancet (London, England)* (2022) 400(10366):1869-81. Epub 2022/11/11. doi: 10.1016/s0140-6736(22)02033-5.
9. Rosenstock J, Frias J, Jastreboff AM, Du Y, Lou J, Gurbuz S, et al. Retatrutide, a Gip, Glp-1 and Glucagon Receptor Agonist, for People with Type 2 Diabetes: A Randomised, Double-Blind, Placebo and Active-Controlled, Parallel-Group, Phase 2 Trial Conducted in the USA. *Lancet (London, England)* (2023) 402(10401):529-44. doi: 10.1016/S0140-6736(23)01053-X.
10. Rosenstock J, Wysham C, Frías JP, Kaneko S, Lee CJ, Fernández Landó L, et al. Efficacy and Safety of a Novel Dual Gip and Glp-1 Receptor Agonist Tirzepatide in Patients with Type 2 Diabetes (Surpass-1): A Double-Blind, Randomised, Phase 3 Trial. *Lancet (London, England)* (2021) 398(10295):143-55. doi: 10.1016/S0140-6736(21)01324-6.
11. Frias JP, Nauck MA, Van J, Kutner ME, Cui X, Benson C, et al. Efficacy and Safety of Ly3298176, a Novel Dual Gip and Glp-1 Receptor Agonist, in Patients with Type 2 Diabetes: A Randomised, Placebo-Controlled and Active Comparator-Controlled Phase 2 Trial. *Lancet (London, England)* (2018) 392(10160):2180-93. doi: 10.1016/S0140-6736(18)32260-8.
12. Garvey WT, Frias JP, Jastreboff AM, le Roux CW, Sattar N, Aizenberg D, et al. Tirzepatide Once Weekly for the Treatment of Obesity in People with Type 2 Diabetes (Surmount-2): A Double-Blind, Randomised, Multicentre, Placebo-Controlled, Phase 3 Trial. *Lancet (London, England)* (2023) 402(10402):613-26. doi: 10.1016/S0140-6736(23)01200-X.
13. Aronne LJ, Sattar N, Horn DB, Bays HE, Wharton S, Lin W-Y, et al. Continued Treatment with Tirzepatide for Maintenance of Weight Reduction in Adults with Obesity: The Surmount-4 Randomized Clinical Trial. *Jama* (2024) 331(1):38-48. doi: 10.1001/jama.2023.24945.

14. Alba M, Yee J, Frustaci ME, Samtani MN, Fleck P. Efficacy and Safety of Glucagon-Like Peptide-1/Glucagon Receptor Co-Agonist Jnj-64565111 in Individuals with Obesity without Type 2 Diabetes Mellitus: A Randomized Dose-Ranging Study. *Clinical obesity* (2021) 11(2):e12432. doi: 10.1111/cob.12432.
15. Jastreboff AM, Kaplan LM, Frías JP, Wu Q, Du Y, Gurbuz S, et al. Triple-Hormone-Receptor Agonist Retatrutide for Obesity - a Phase 2 Trial. *The New England journal of medicine* (2023) 389(6):514-26. doi: 10.1056/NEJMoa2301972.
16. Sanyal AJ, Bedossa P, Fraessdorf M, Neff GW, Lawitz E, Bugianesi E, et al. A Phase 2 Randomized Trial of Survodutide in Mash and Fibrosis. *The New England journal of medicine* (2024) 391(4):311-9. doi: 10.1056/NEJMoa2401755.
17. Zhao L, Cheng Z, Lu Y, Liu M, Chen H, Zhang M, et al. Tirzepatide for Weight Reduction in Chinese Adults with Obesity: The Surmount-Cn Randomized Clinical Trial. *Jama* (2024) 332(7):551-60. doi: 10.1001/jama.2024.9217.
18. Yazawa R, Ishida M, Balavarca Y, Hennige AM. A Randomized Phase I Study of the Safety, Tolerability, Pharmacokinetics and Pharmacodynamics of Bi 456906, a Dual Glucagon Receptor/Glucagon-Like Peptide-1 Receptor Agonist, in Healthy Japanese Men with Overweight/Obesity. *Diabetes, obesity & metabolism* (2023) 25(7):1973-84. doi: 10.1111/dom.15064.
19. Ji L, Jiang H, Cheng Z, Qiu W, Liao L, Zhang Y, et al. A Phase 2 Randomised Controlled Trial of Mazdutide in Chinese Overweight Adults or Adults with Obesity. *Nature communications* (2023) 14(1):8289. doi: 10.1038/s41467-023-44067-4.
20. Jastreboff AM, Aronne LJ, Ahmad NN, Wharton S, Connery L, Alves B, et al. Tirzepatide Once Weekly for the Treatment of Obesity. *The New England journal of medicine* (2022) 387(3):205-16. doi: 10.1056/NEJMoa2206038.
21. Véniant MM, Lu S-C, Atangan L, Komorowski R, Stanislaus S, Cheng Y, et al. A Gipr Antagonist Conjugated to Glp-1 Analogues Promotes Weight Loss with Improved Metabolic Parameters in Preclinical and Phase 1 Settings. *Nature metabolism* (2024) 6(2):290-303. doi: 10.1038/s42255-023-00966-w.
22. le Roux CW, Steen O, Lucas KJ, Startseva E, Unseld A, Hennige AM. Glucagon and Glp-1 Receptor Dual Agonist Survodutide for Obesity: A Randomised, Double-Blind, Placebo-Controlled, Dose-Finding Phase 2 Trial. *The lancet Diabetes & endocrinology* (2024) 12(3):162-73. doi: 10.1016/S2213-8587(23)00356-X.
23. Ji L, Gao L, Jiang H, Yang J, Yu L, Wen J, et al. Safety and Efficacy of a Glp-1 and Glucagon Receptor Dual Agonist Mazdutide (Ibi362) 9 Mg and 10 Mg in Chinese Adults with Overweight or Obesity: A Randomised, Placebo-Controlled, Multiple-Ascending-Dose Phase 1b Trial. *EClinicalMedicine* (2022) 54:101691. doi: 10.1016/j.eclinm.2022.101691.
24. Ji L, Jiang H, An P, Deng H, Liu M, Li L, et al. Ibi362 (Ly3305677), a Weekly-Dose Glp-1 and Glucagon Receptor Dual Agonist, in Chinese Adults with Overweight or Obesity: A Randomised, Placebo-Controlled, Multiple Ascending Dose Phase 1b Study. *EClinicalMedicine* (2021) 39:101088. doi: 10.1016/j.eclinm.2021.101088.
